# Next-Generation Sequencing Analysis of the Human TCRγδ+ T-Cell Repertoire Reveals Shifts in Vγ- and Vδ-Usage in Memory Populations upon Aging

**DOI:** 10.3389/fimmu.2018.00448

**Published:** 2018-03-06

**Authors:** Martine J. Kallemeijn, François G. Kavelaars, Michèle Y. van der Klift, Ingrid L. M. Wolvers-Tettero, Peter J. M. Valk, Jacques J. M. van Dongen, Anton W. Langerak

**Affiliations:** ^1^Laboratory for Medical Immunology, Department of Immunology, Erasmus University Medical Center, Rotterdam, Netherlands; ^2^Department of Hematology, Erasmus University Medical Center, Rotterdam, Netherlands

**Keywords:** TCRγδ+, development, aging, repertoire, next-generation sequencing

## Abstract

Immunological aging remodels the immune system at several levels. This has been documented in particular for the T-cell receptor (TCR)αβ+ T-cell compartment, showing reduced naive T-cell outputs and an accumulation of terminally differentiated clonally expanding effector T-cells, leading to increased proneness to autoimmunity and cancer development at older age. Even though TCRαβ+ and TCRγδ+ T-cells follow similar paths of development involving V(D)J-recombination of TCR genes in the thymus, TCRγδ+ T-cells tend to be more subjected to peripheral rather than central selection. However, the impact of aging in shaping of the peripheral TRG/TRD repertoire remains largely elusive. Next-generation sequencing analysis methods were optimized based on a spike-in method using plasmid vector DNA-samples for accurate TRG/TRD receptor diversity quantification, resulting in optimally defined primer concentrations, annealing temperatures and cycle numbers. Next, TRG/TRD repertoire diversity was evaluated during TCRγδ+ T-cell ontogeny, showing a broad, diverse repertoire in thymic and cord blood samples with Gaussian CDR3-length distributions, in contrast to the more skewed repertoire in mature circulating TCRγδ+ T-cells in adult peripheral blood. During aging the naive repertoire maintained its diversity with Gaussian CDR3-length distributions, while in the central and effector memory populations a clear shift from young (Vγ9/Vδ2 dominance) to elderly (Vγ2/Vδ1 dominance) was observed. Together with less clear Gaussian CDR3-length distributions, this would be highly suggestive of differentially heavily selected repertoires. Despite the apparent age-related shift from Vγ9/Vδ2 to Vγ2/Vδ1, no clear aging effect was observed on the Vδ2 invariant T nucleotide and canonical Vγ9–Jγ1.2 selection determinants. A more detailed look into the healthy TRG/TRD repertoire revealed known cytomegalovirus-specific TRG/TRD clonotypes in a few donors, albeit without a significant aging-effect, while *Mycobacterium tuberculosis*-specific clonotypes were absent. Notably, in effector subsets of elderly individuals, we could identify reported TRG and TRD receptor chains from TCRγδ+ T-cell large granular lymphocyte leukemia proliferations, which typically present in the elderly population. Collectively, our results point to relatively subtle age-related changes in the human TRG/TRD repertoire, with a clear shift in Vγ/Vδ usage in memory cells upon aging.

## Introduction

Immunological aging, also referred to as immunosenescence, is a complex phenomenon consisting of senescence and exhaustion processes, which are characterized by different functional and marker expression profiles (1, 2). Immunosenescence acts on different levels in the immune system, e.g., reduced antigen-specific responses (3), thymic shrinkage, and a significantly reduced naive T-cell output (3–5), convergence of the innate and adaptive immunity (6), and ultimately T-cell exhaustion (1). Immunosenescence is believed to play a major role in shaping of the antigen receptor repertoire of T-cells.

T-cells develop in the thymus, where they undergo commitment, rearrangement, selection and maturation processes. The main event during T-cell development is the rearrangements of the variable (V), diversity (D), and joining (J) genes of the T-cell receptor (TR) loci, in order to establish a large diversity of antigen receptors (7, 8). Two main types of T-cells are generated; first, TCRγδ+ thymocytes, through early TR delta and gamma (TRD, TRG) rearrangements, then followed by TCRαβ+ thymocytes upon TR beta and alpha (TRB, TRA) rearrangements (8). TCRαβ+ thymocytes undergo positive selection through TCR signaling to subsequently mature into functional T-cells, followed by negative selection in order to eliminate self-reactive T-cell precursors (9). In contrast, TCRγδ+ thymocytes do not undergo positive and/or negative selection in the thymus (10), but extrathymic development and peripheral (antigenic) selection of TCRγδ+ T-cells have been described (11).

TCRγδ+ T-cells appear to be the first functional population of circulating T lymphocytes in both murine and human peripheral blood (PB) [reviewed in Ref. (12)]. In the human fetal and neonatal situation these functional circulating TCRγδ+ T-cells mainly concern Vδ1+ cells. Readily after birth and during further development to adulthood a switch occurs in the circulating TCRγδ+ T-cell population with the number of Vδ1+ cells decreasing and Vγ9/Vδ2 cells becoming the predominant TCRγδ+ T-cell types (13). This process is believed to be the result of peripheral antigenic selection, exemplified by the presence of an invariant T nucleotide in the majority of the selected Vδ2–Jδ1 rearrangements (13–15). Furthermore, epitopes from pathogens or other antigens that could stimulate and select TCRγδ+ T-cell types have been described: *Mycobacterium tuberculosis* has been found to be a major stimulator of Vγ9/Vδ2 cells in both infected lungs and PB (16), whereas non-Vγ9/Vδ1 cells are known to be stimulated by viruses, such as cytomegalovirus (CMV) (17, 18) and Epstein-Bar virus (EBV) (19). TCRγδ+ T-cells do not only recognize antigens *via* their receptor, but they also respond to lipid antigens presented on CD1d-molecules, and that are associated with stress, inflammation and cancer [reviewed by Ref. (20)]. Most TCRγδ+ T-cells recognizing these CD1d-lipid antigen complexes are Vδ1 or Vδ3 cells, commonly located in the gut (21). TCRγδ+ T-cells can also recognize butyrophilins, tumor-antigens, endothelial antigens, antigen-presenting cells, and Toll-like receptors [reviewed in Ref. (22)], all of which are postulated to contribute to shaping of the TCRγδ+ T-cell repertoire.

TCRγδ+ T-cell recognition and selection has been mostly described in the context of the developing immune system from fetus to neonate and adulthood, but—contrary to the TCRαβ+ T-cell repertoire—effects of aging on the TCRγδ+ T-cell repertoire have not been extensively addressed. Since it has been found that TCRγδ+ T-cells follow the classical aging model as found in mainly CD8+ TCRαβ+ T-cells (23), we hypothesized that the naive mature TCRγδ+ T-cell repertoire would depict a broad spectrum of rearrangements and that it would show a more skewed pattern during further development from neonates to young adults and eventually elderly individuals. Furthermore, in view of the fact that T-cell large granular lymphocyte (LGL) leukemia typically presents as a proliferation of effector cells in elderly, we were interested to compare our TRG/TRD repertoire findings to the LGL clonal repertoire. To this end, we investigated the developing and aging TRG/TRD repertoire in TCRγδ+ T-cell subsets, using an optimized experimental next-generation sequencing (NGS) procedure to minimize technical biases of PCR-based methods. Our data show subset- and donor-specific TRG/TRD repertoires, suggestive of selection, with significant differences in the combinatorial repertoire in especially memory populations between young and elderly individuals. When looking closer into TRG/TRD clonotypes, TCRγδ+ T-LGL leukemia receptor chains could be traced in especially the effector subsets of elderly individuals, which would fit the current idea that TCRγδ+ T-LGL leukemia cells originate from the normal healthy antigen-experienced TCRγδ+ T-cells.

## Materials and Methods

### Subjects and Materials

Blood from healthy blood donors from Sanquin Blood Bank (Amsterdam, The Netherlands) in the age range of 20–35 years (young adults, *N* = 11) and 56–70 years (elderly, *N* = 12) was used upon written informed consent at the blood bank (project number NVT0012.01) and anonymized for further use. The maximum age to donate blood is 70 years. Healthy neonatal cord blood (CB) was obtained postpartum or after Caesarian section through collaboration and upon written informed consent at the Departments of Obstetrics and Hematology. CB was drawn using CB Collect bags containing citrate phosphate dextrose solution as anticoagulant. Thymic lobes were removed upon heart surgery in individuals under the age of two years upon written informed consent from parents. Both CB and thymus material was obtained under Medical Ethics Committee approval (project number hmPOO2004-003). Whole thymic material was sliced and prepared prior to cryopreservation. Peripheral blood mononuclear cells (PBMCs) and cord blood mononuclear cells (CBMCs) were obtained through Ficoll density gradient separation. Isolated PBMCs, CBMCs, and thymocytes were cryopreserved in Iscove’s Modified Dulbecco’s Medium (Lonza, Basel, Switzerland) with dimethyl sulfoxide and stored in vials at −180°C until further use. All studies were conducted in accordance with the principles of the Declaration of Helsinki.

### Cell Sorting

Cryopreserved material was thawed and sorted using CD3, CD45, TCRαβ, TCRγδ, CD45RA, CD45RO, CD27, and CD197 antibodies (Table S1 in Supplementary Material) to obtain TCRγδ+ naive (CD45RA+ CD27+ CD197 +), central memory (CD45RA-CD45RO+ CD27+ CD197+), effector memory (Temro population defined as CD45RA-CD45RO+ CD27− CD197−), and effector (Temra population, CD45RA+ CD27− CD197−) T-cells (Figure S1 in Supplementary Material). Cell sorting was performed with FACS Aria I and III instruments (BD Biosciences, San Jose, CA, USA).

### DNA Isolation

Following isolation, cells were lysed and subjected to DNA isolation using the DNA/RNA/miRNA AllPrepKit according to the manufacturer’s protocol (Qiagen, Hilden, Germany). DNA concentration and quality (A260/A280 absorption ratio) were determined by Nanodrop measurements (Thermo Fischer Scientific, Waltham, MA, USA).

### Primer Design

Primers for cloning and Illumina-based sequencing were largely based on those reported in BIOMED-2 assays (24). The Vδ3 primer was redesigned to better fit amplicon length of PCR products generated with the existing Vδ1 and Vδ2 primers. The Jγ1.2 primer was newly designed, as this primer was not included in the BIOMED-2 TRG assay. Vγ1F and Jγ1.3/2.3 primers were adjusted compared with the BIOMED-2 protocol (Table S2 in Supplementary Material). Primers were adapted for Illumina-based sequencing by adding Illumina forward (5′-ACACTCTTTCCCTACACGACGCTCTTCCGATCT-3′) and reverse (5′-TCGCGAGTTAATGCAACGATCGTCGAAATTCGC-3′) overhang adaptor sequences to the respective primers. The second PCR, by means of these overhang adaptor sequences, attaches sample-specific dual indices for sample identification and Illumina sequencing adaptors using primers from the Illumina TruSeq Custom Amplicon Index Kit (Illumina, San Diego, CA, USA).

### Plasmid Pool Preparation

Primer validation and titration was done using plasmid vectors with cloned TRD and TRG gene rearrangements. All possible V–J gene combinations were PCR amplified and cloned from immature T-cell lines (25) and thymus DNA into the pGEM T-Easy vector in a 3:1 insert:vector ratio according to the manufacturer’s protocol (Promega, Madison, WI, USA). Composition of the plasmid pools is summarized in Table S3 in Supplementary Material.

### Assay Optimization Experiments

PCRs were first tested in singleplex and multiplex settings with varying primer concentrations, annealing temperatures and PCR cycle numbers. Each initial PCR mix contained GeneAmp PCR Buffer II (1×), magnesium chloride (2.5 mM), dNTPs (2.0 mM), and AmpliTaqGold (1 U) (Thermo Fischer Scientific). Total forward and reverse primer(s) amounts were generally 10 pmol. The PCR protocol was largely based on the BIOMED-2 publication (23), with varying annealing temperatures (Tm = 58/59/60/62) and different numbers of cycles (20 and 25 cycles). Primer concentration adjustment, and optimization of annealing temperatures and number of PCR cycles were based on the results of iterative optimization experiments as summarized in Table S4 and Figures S2 and S3 in Supplementary Material.

### Amplicon Preparation

Amplicons from the first step PCR were purified using the Agencourt AMPure XP bead purification kit (Beckman Coulter, Fullerton, CA, USA), whereafter concentrations were measured with the Quant-iT PicoGreen dsDNA Assay Kit (Thermo Fischer Scientific), after which the amplicons were adjusted to similar concentrations. The second step PCR was performed with primers from the Illumina TruSeq Custom Amplicon Index Kit (Illumina) using the KAPA HiFi HotStart PCR Kit (Kapa Biosystems, Wilmington, MA, USA). Second PCR amplicons were evaluated *via* agarose gel electrophoresis or PicoGreen concentration measure-ment. Library pool preparation was subsequently performed based on the gel image or PicoGreen measurement results. The library pool was further purified with Agencourt AMPure XP beads and normalized for Illumina-based sequencing, according to the manufacturer’s protocol (Illumina).

### Next-Generation Sequencing

Paired-end NGS (2 × 221 bp) was performed on the MiSeq platform (Illumina, San Diego, CA, USA) with the use of an Illumina MiSeq Reagent Kit V3, according to the manufacturer’s protocol (Illumina).

### Bioinformatic Data Analysis

Illumina NGS data were obtained in FASTQ format. Paired-end reads were combined using the FASTQ-join tool in the Erasmus MC Galaxy Server (26), with the use of usegalaxy.org (27–29) converted from FASTQ to FASTA with the converter tool (30). Sequencing annotations were made *via* the IMGT High V-quest database (31–34). Calculation of the clonality score for multiple replicates was based on the algorithm described by Boyd et al. (35). Clonal type definition was based on V and J gene usage and CDR3-region at the nucleotide level. Rearrangements were visualized using Circoletto plots [www.circos.ca (36)]. CDR3 amino acid compositions were visualized using WebLogo online tool [www.weblogo.berkeley.edu (37, 38)].

The NGS TRG-TRD data set has been submitted to the BioProject repository (BioProjectID: PRJNA434217, submissionID SUB3660187; http://www.ncbi.nlm.nih.gov/bioproject/434217). Sequencing details can be accessed through SRA database accession SRP133150 (https://www.ncbi.nlm.nih.gov/sra/SRP133150).

### Statistical Analysis

Data were checked for normal distributions using the Hartigan’s Dip Test Statistic for Unimodality package (39–41) in R version 3.4.1 (42). All statistical analyses were performed with Prism 5 (GraphPad, La Jolla, CA, USA).

## Results

### Multiplex PCR Assay Fine-Tuning Leads to an Optimized, Bias-Free NGS Assay for Reliable Quantification of the TRG/TRD Repertoire

**Figure 1:**
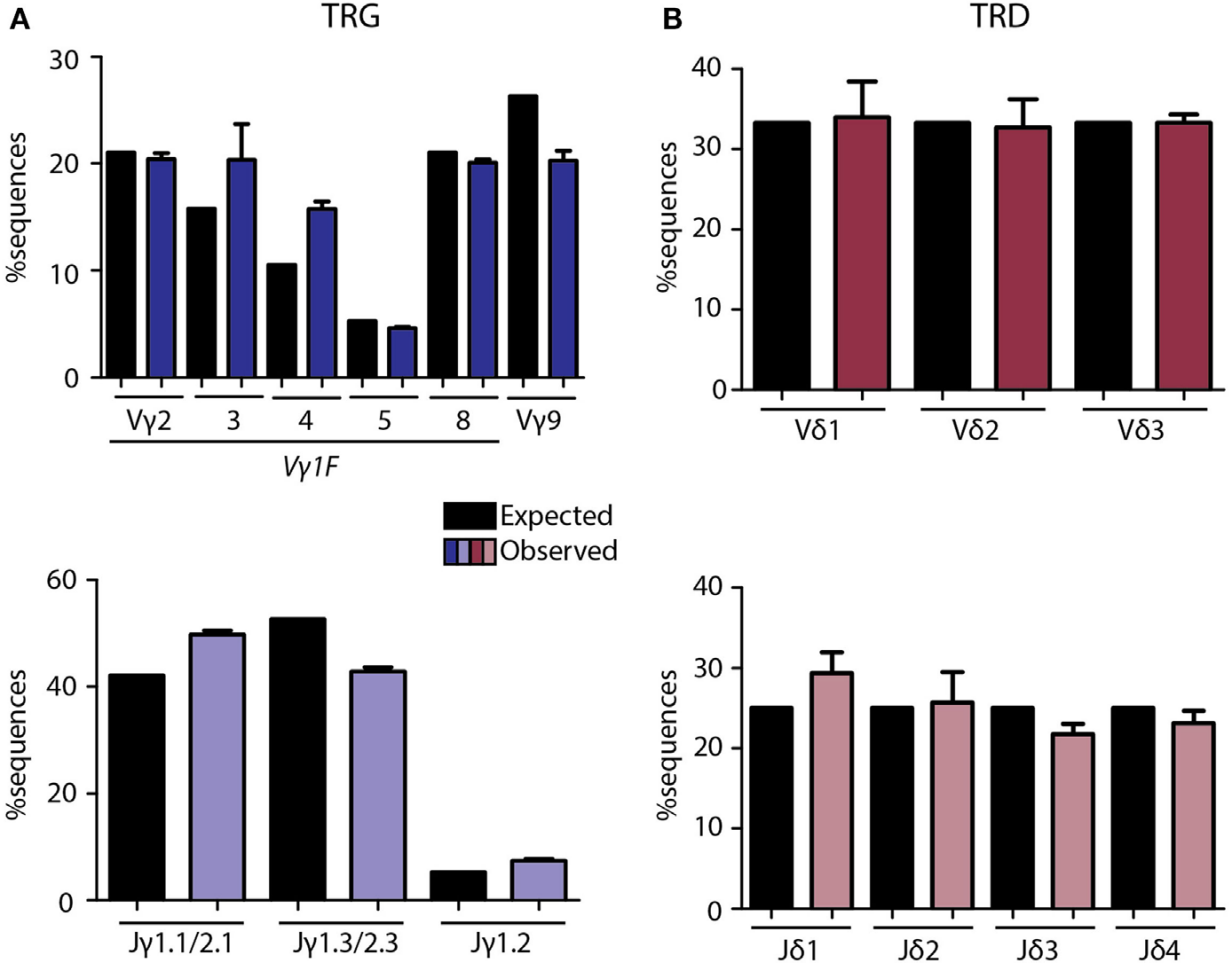
Technical optimization of next-generation sequencing assays for TRG/TRD loci. Multiplex PCR assays were optimized with balanced primer concentrations, annealing temperatures, and cycle numbers as summarized in Table S3 in Supplementary Material. Plasmid pools were used as spike-in samples to determine the percentage expected sequences per V and J gene vs. the observed percentage after sequencing (Table S2 in Supplementary Material). Expected percentages are indicated in black bars, observed percentages are indicated in colored bars. TRG assays showed high overlap between frequencies of expected and observed sequences for Vγ and Jγ **(A)** genes, with some variation due to single primers covering multiple genes (Vγ1F covering Vγ2-8, Jγ1.1/2.1 covering Jγ1.1 and Jγ2.1, and Jγ1.3/2.3 covering Jγ1.3 and Jγ2.3). TRD assays showed nearly similar percentages of expected and observed sequences for Vδ and Jδ **(B)** genes. Error bars represent SD of PCR replicates (*N* = 4).

The multiplex PCR assay to be analyzed by NGS was optimized and fine-tuned for more accurate quantification and receptor diversity analysis of the TRG and TRD loci using a diverse set of artificial DNA spike-in samples and primer concentration titration experiments (Tables S4 and S5 and Figures S2 and S3 in Supplementary Material). Each artificial DNA sample, represented by plasmid vector DNA, contained a mixture of known V(D)J rearrangements, cloned from either immature T-cell lines or thymus DNA in equimolar proportions (Table S4 in Supplementary Material). After several rounds of fine-tuning (Figures S2 and S3 in Supplementary Material), and repeated technical validation with plasmid spike-in pools we established the most optimal PCR conditions for both TRG (Figure 1A) and TRD (Figure 1B) multiplex assays in view of unbiased NGS data. Remaining small differences between observed and expected read frequencies are introduced by chance in the PCR reaction and/or due to inevitable interassay variation. These were reduced to a minimum by using four replicates for each sample, which included four differently pipetted mixes to reduce pipetting bias and the use of four different PCR machines to reduce machine-dependent bias. These optimization experiments resulted in variable primer concentrations and defined annealing temperatures and cycle numbers for the TRG and TRD multiplex PCR reactions (Table S5 in Supplementary Material).

### The TRG/TRD Repertoire Is Diverse in Immature Thymus and CB, and More Skewed in Mature Circulating TCRγδ+ T-Cells

**Figure 2:**
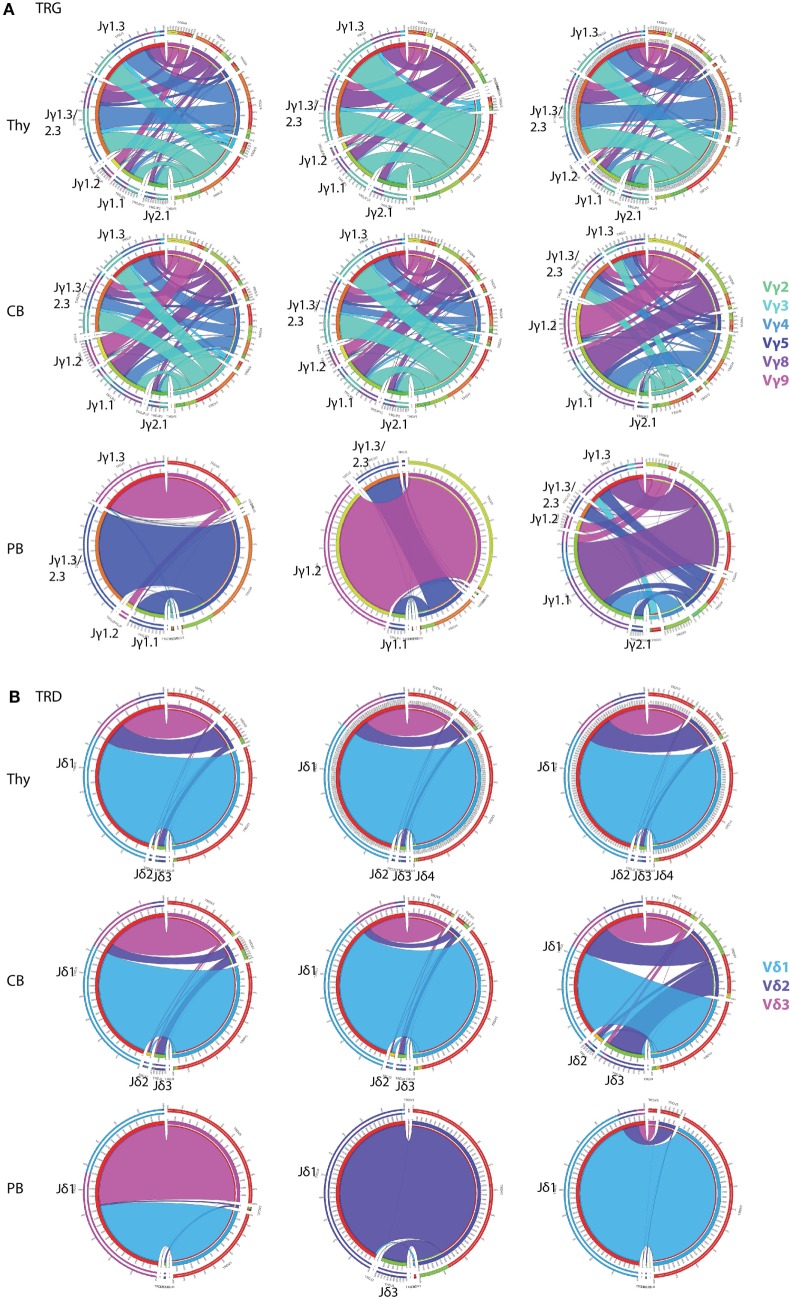
Circoletto visualization of the TRG/TRD repertoire during ontogeny. Optimized multiplex PCR next-generation sequencing assays were applied on total TCRγδ+ T-cells sorted from thymus (Thy), neonatal cord blood (CB), and adult peripheral blood (PB). TRG assays showed high repertoire diversity in both Thy and CB samples, with low interindividual variation, while adult PB samples showed individual-specific repertoire patterns with less receptor diversity **(A)**. TRD assays showed high dominance of Vδ1 (light blue bars), which was also observed in CB samples, both with low intersample variation. Adult PB samples showed donor-specific patterns with sometimes skewing toward Vδ2 and even Vδ3 **(B)**. Three representative samples of each samples type are visualized: Thy04-10, Thy05-13, Thy10-03, CB2, CB3, CB4, PB30, PB31, and PB50. Plots were made using the Circoletto online software tool [http://www.circos.ca (35)]. Each band represents a V–J rearrangement, with colors based on V-gene usage.

In order to determine changes in TCRγδ+ T-cell repertoire in healthy individuals, we first investigated TRG/TRD repertoire diversity during ontogeny using purified TCRγδ+ T-cells from different compartments, i.e., thymus (Thy) and neonatal CB. In addition, we sequenced the total mature TCRγδ+ T-cell population of healthy adult PB samples. TRG rearrangements in Thy and CB samples were highly diverse (Figure 2A, upper two rows), and the intersample variation of especially Thy samples was low, in keeping with the non-selected character of the TCRγδ+ T-cells in these compartments. These findings were in strong contrast to PB samples, which showed a high level of skewing and predominance of certain receptors (including Vγ9–Jγ1.2 sequences) were observed (Figure 2A, bottom row), albeit with high inter-individual differences, illustrating the dominant role of (antigenic) selection. TRD diversity was less apparent, although in Thy and CB samples (Figure 2B, upper two rows) all three predominant Vδ-genes were identified. Again, intersample variation was low, illustrating the non-selected character of Thy and CB cells. Intersample variation was more evident for the PB samples (Figure 2B, bottom row), with predominance of Vδ2 usage, but also Vδ3 usage in some cases, reflecting different types of (antigenic) selection between individuals.

Collectively, these data confirmed our hypothesis of a broad and diverse TRG/TRD repertoire in the immature Thy and CB samples and a more skewed TRG/TRD repertoire in mature circulating TCRγδ+ T-cells in adults, thereby validating our optimized multiplex PCR-based TRG/TRD NGS assays.

### Upon Aging Memory TCRγδ+ T-Cells Show Shifts in V-Gene Usage, Whereas Naive and Effector Populations Do Not

As it has become evident that aging plays a major role in shaping the elderly immune system (43), we next evaluated the role of aging on the combinatorial TRG/TRD repertoire. To this end, we sorted TCRγδ+ T-cells from healthy young (*N* = 11; age range 20–35) and elderly (*N* = 12; age range 56–70) individuals into four subsets: naive (CD45RA+ CD45RO− CD27+ CD197+), central memory (CD45RA− CD45RO+ CD27+ CD197+), effector memory (Temro; CD45RA− CD45RO+ CD27− CD197−), and effector (Temra; CD45RA+ CD45RO− CD27− CD197−) TCRγδ+ T-cells. Subset distributions of young and elderly individuals (Figure S4 in Supplementary Material) correlated with those from our previous aging study, from which it is known that TCRγδ+ T cells show little CCR7 expression fitting with low absolute and relative numbers of naive and central memory cells (44) (Table S6 in Supplementary Material). Even though the spectrum of V–J combinations for both TRG and TRD varied in a donor-specific way between individuals (Figure S5 in Supplementary Material), the overall TRG/TRD combinatorial diversity appeared to be mostly determined by differences in Vγ/Vδ usage rather than Jγ/Jδ gene usage.

**Figure 3:**
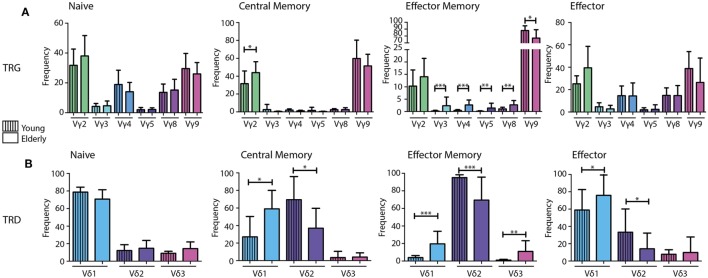
V gene diversity in the combinatorial TRG/TRD repertoire of different TCRγδ+ T-cell subsets from young and elderly individuals. V-gene usage per subset visualized in bar graphs indicating a diverse repertoire in naive and effector subsets of both young and elderly, with skewing toward Vγ2 and Vγ9 in memory populations **(A)**. Naive populations showed diversity in Vδ usage with Vδ1 dominance in young and elderly. Young individuals showed relatively higher Vδ2 usage in especially the memory and effector subsets, whereas elderly individuals showed clear Vδ1 dominance in all subsets, except for effector memory cells **(B)**. Median V-gene frequencies of productive sequences with SD bars were indicated in bar graphs for young (*N* = 11) and elderly (*N* = 12) individuals. Color legends are indicated in the figure. Statistical significance was tested using the Mann–Whitney *U*-test. Level of significance is indicated in the plots: *p < 0.05; **p < 0.01; ***p < 0.0001.

Naive TCRγδ+ T-cells of both young and elderly individuals showed a relatively diverse TRG repertoire, which was in strong contrast to (central and effector) memory TCRγδ+ T-cells that showed dominant Vγ9 gene usage. Effector TCRγδ+ T-cells of both age groups were more diverse again. Of note, significant differences between young and elderly could be observed in mainly the memory populations, as reflected by a significantly higher Vγ2-usage in central memory TCRγδ+ T-cells in elderly, as well as significantly lower Vγ2-8-usage and significantly higher Vγ9 gene usage in effector memory cells of elderly (Figure 3A).

When comparing TRD combinatorial profiles in the different subsets between young and elderly individuals, significantly higher Vδ1 and significantly lower Vδ2 gene usage was observed in memory populations of elderly individuals. This effect was also observed in the effector population. In the effector memory cells of elderly Vδ3 gene usage was also significantly higher (Figure 3B).

Overall, these data show clear differences in the TRG/TRD combinatorial repertoire between naive TCRγδ+ T-cells on the one hand and especially memory TCRγδ+ T-cells on the other hand. Notably, the clear dominance of Vγ9 and Vδ2 usage in memory and effector TCRγδ+ T-cells in young individuals was less prominent in elderly individuals, who on average showed significant shifts toward more Vγ2 and Vδ1 gene usage in addition to Vγ9 and Vδ2. Most significant differences between young and elderly were identified in central and effector memory populations.

### The TRG/TRD Junctional Region Repertoire Shows Signs of Selection in Memory and Effector Cell Populations of both Young and Old Individuals

**Figure 4:**
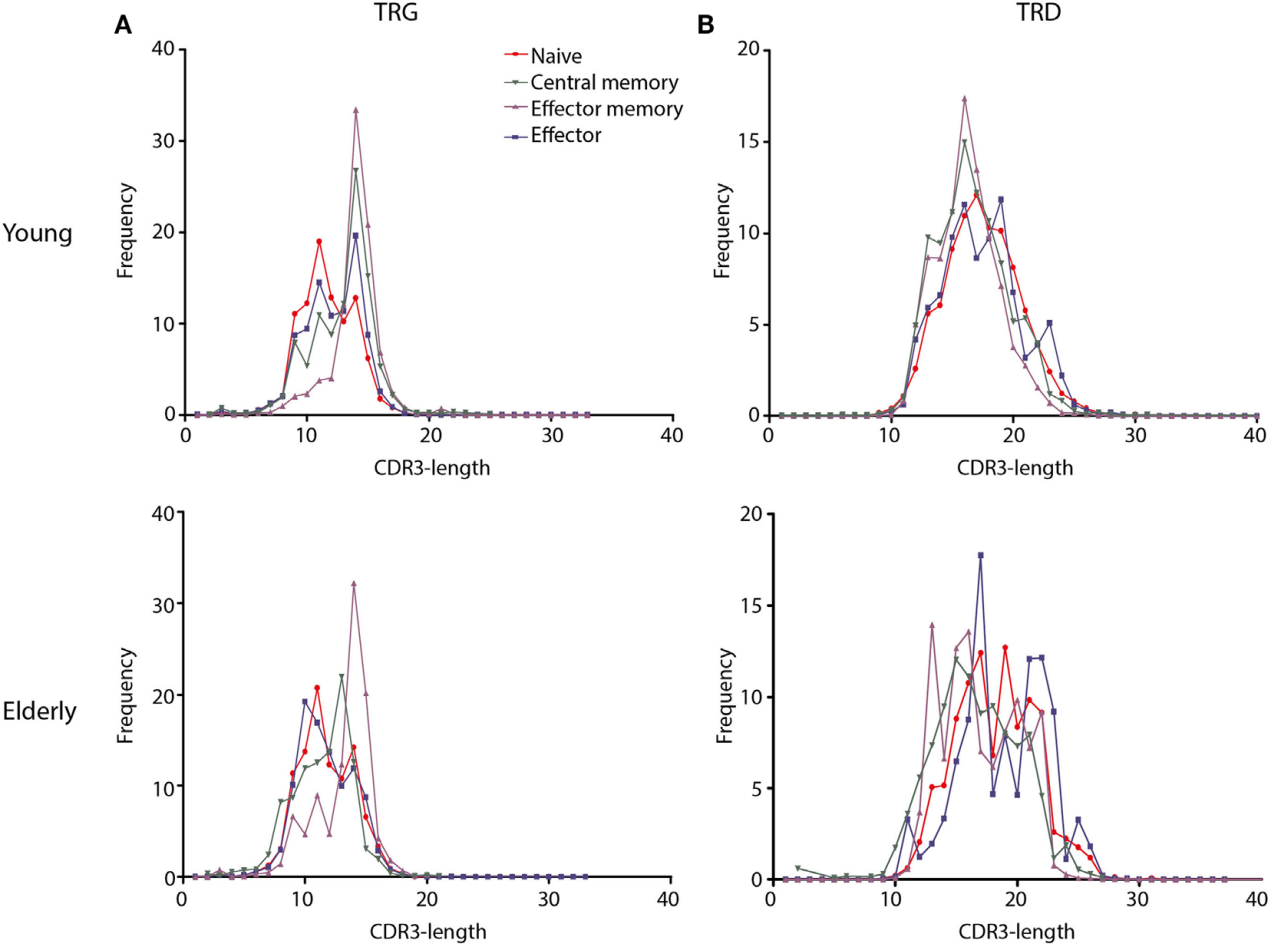
CDR3-length distributions of TCRγδ+ T-cell subsets from young and elderly individuals. Frequencies of CDR3-lengths from different TCRγδ+ T-cell subsets are summarized per T-cell receptor chain and age group. Naive subsets (red lines) showed distributions resembling Gaussian profiles, while memory subsets showed dominant peaks suggestive of selection and receptor skewing (green and purple lines). TRG CDR3-lengths showed similar distributions between young and elderly **(A)**, TRD CDR3-lengths in young individuals showed a Gaussian profile in the naive subset and more skewing in memory and effector subsets, whereas in elderly individuals all subsets showed dominant peaks **(B)**. Mean frequencies per subset were indicated for young (*N* = 11) and elderly (*N* = 12) individuals. Data normality was tested using the diptest package in R.

For a more detailed view of the TRG/TRD repertoire, we then studied CDR3-regions, which reflect the most relevant antigen-binding part of the antigen receptors. These CDR3-length distributions are indicative of the junctional repertoire. TRG/TRD CDR3-length distributions of Thy TCRγδ+ T-cells showed Gaussian profiles, just like the TRD CDR3-length distributions of CB TCRγδ+ T-cells; TRG CDR3-lengths of CB TCRγδ+ T-cells showed less clear Gaussian distributions and more prominent peaks, probably reflecting low-level selection (Figure S6 in Supplementary Material). The effect of selection became even more evident in adult individuals; naive TCRγδ+ T-cells showed mostly Gaussian CDR3 profiles, in contrast to memory and effector TCRγδ+ T-cells of young individuals, which showed dominant peaks for both the TRG and TRD CDR3-regions (Figures 4A,B). Elderly individuals did not show clear Gaussian profiles, and even prominent peaks in all subsets, thus reflecting a more heavily selected repertoire (Figures 4A,B). The average TRG and TRD CDR3-lengths were not markedly different between young and elderly individuals.

### TRG Canonical and TRD Invariant T Selection Determinants are Detectable in Normal TCRγδ+ T-Cells but Do Not Increase upon Aging

**Figure 5:**
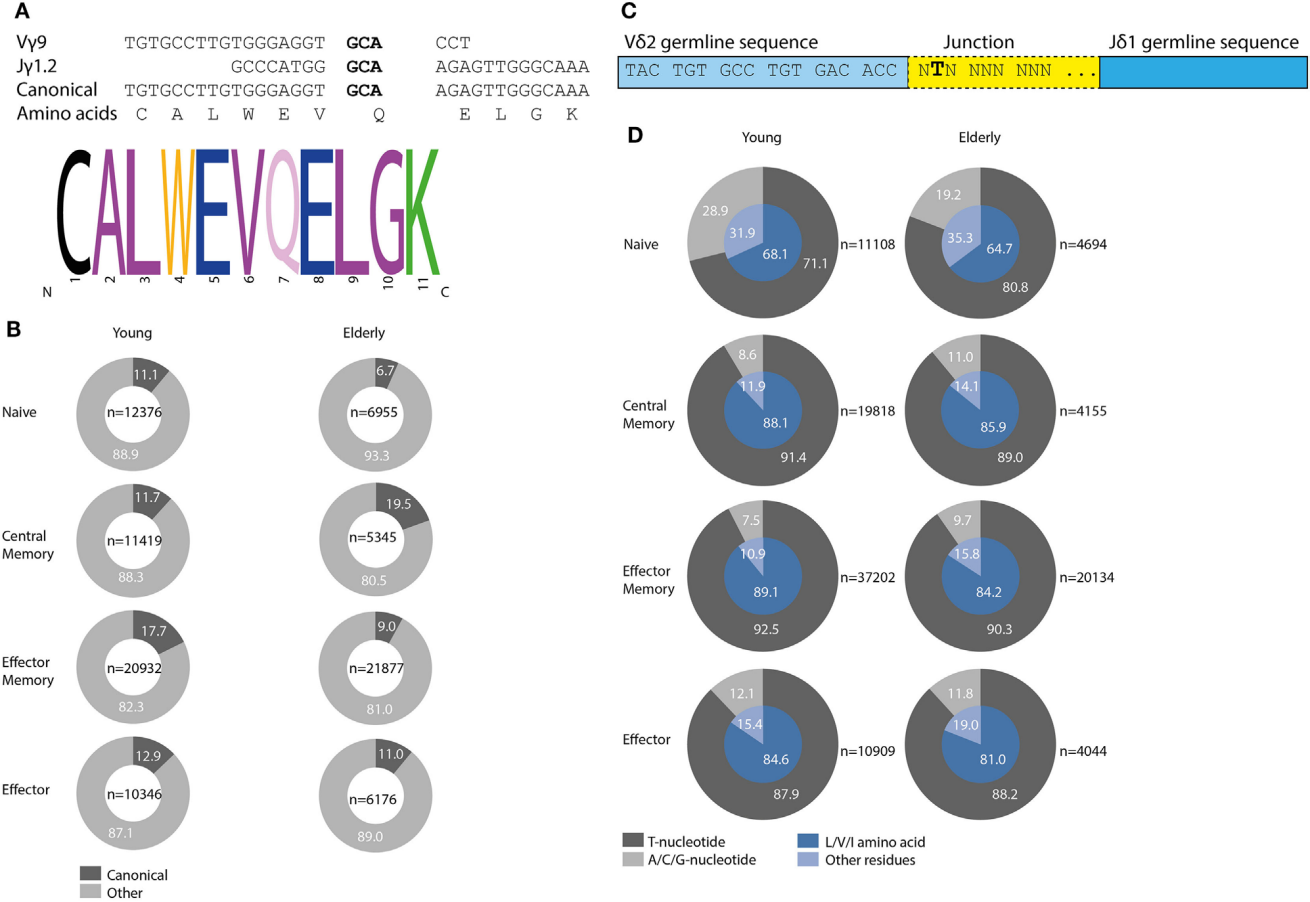
Canonical Vγ9–Jγ1.2 sequences and Vδ2–Jδ1 invariant T selection determinant in young and elderly healthy individuals. GCA sequence in bold mediates preferential recombination of Vγ9 with Jγ1.2, resulting in specific CDR3 length and amino acid composition, adapted from Ref. (45) **(A)**. Frequencies of all productive Vγ9–Jγ1.2 sequences containing the canonical sequence per age group and subset marked in dark gray. In light gray Vγ9–Jγ1.2 rearrangements with other, non-canonical sequences are indicated. Numbers of total Vγ9–Jγ1.2 sequences are indicated in center of plots **(B)**. The invariant T selection determinant is located at the relative second position of the first codon in the junction of Vδ2–Jδ1 rearrangements, adapted from Ref. (13) **(C)**. Frequencies of invariant T selection determinant in young and elderly subsets (dark gray part outer ring), leading to L, V, or I amino acid residues (inner blue pie chart, dark blue part). Absolute number of unique productive Vδ2–Jδ1 sequences indicated next to plots **(D)**.

During development selection of TCRγδ+ T-cells is known to be associated with so-called selection determinants, which represent molecular fingerprints in the CDR3-regions of TRG and TRD chains. In circulating TCRγδ+ T-cells a high frequency of Vγ9–Jγ1.2 recombinations with preferential joining at the GCA sequence has been noted (Figure 5A). We therefore studied this so-called canonical Vγ9–Jγ1.2 rearrangement, characterized by a defined CDR3-length and amino acid composition (Figure 5A), in different subsets of young and elderly healthy controls. Approximately 10–20% of all productive Vγ9–Jγ1.2 rearrangements contained the canonical sequence (Figure 5B). The frequencies of canonical Vγ9–Jγ1.2 sequences did not clearly differ between different subsets in young and elderly (Figure 5B). In TCRγδ-receptors the canonical Vγ9–Jγ1.2 chain is frequently combined with a Vδ2-derived chain, especially resulting from Vδ2–Jδ1 recombination. These Vδ2–Jδ1 rearrangements often contain a so-called invariant T nucleotide, a selection determinant at the relative second position of the first codon of the junctional region (Figure 5C), translating into leucine (L), valine (V), or isoleucine (I) amino acids at that first codon in the junction. The invariant T was observed in all individuals (Figure 5D, outer gray circles), and resulted in L, V or I amino acids at this position (Figure 5D, inner blue pie charts). The invariant T was present at higher frequency in memory and effector subsets compared to naive TCRγδ+ T-cell subsets. On average, invariant T frequencies per subset did not differ much between young and elderly individuals, although the percentage of invariant T-containing sequences at the nucleotide level of naive TCRγδ+ T-cells of young individuals was clearly lower than that of elderly naive TCRγδ+ T-cells (Figure 5D).

Taken together, the most common selection determinants described in TCRγδ+ T-cells (i.e., the Vγ9–Jγ1.2 canonical sequence and the Vδ2–Jδ1 invariant T nucleotide) were readily identified in different TCRγδ+ T-cell subsets in our healthy control cohort, albeit that frequencies did not clearly differ between young and elderly.

### Analysis of TRG/TRD Clonotypes Shows the Presence of TCRγδ+ T-LGL Leukemia-Related Clonotypes in Especially Effector Cells of Elderly

In view of TCRγδ+ T-cell selection processes, we then studied the possible recurrence of specific TRG/TRD clonotypes in the repertoire of young and elderly individuals, as a sign of activated TCRγδ+ T-cell clones. To this end multiple replicates (*N* = 3) of each TCRγδ+ T-cell subset were studied in independent PCR reactions and the number of so-called coincident sequences was determined (35, 46). In all subsets, both young and elderly, the frequency of clonotype sequences found in only one of the replicates was the highest, while the frequencies of coincidences found in two or three replicates were relatively low for both TRG and TRD (Figure S7 in Supplementary Material). When comparing young and elderly, small shifts leading to higher numbers of coincidences in two or three replicates were seen in the latter (Figure S7 in Supplementary Material). We then only focused on the coincidences present in all three replicates, since these sequences best reflect the individuals’ repertoire selection. Especially in the effector memory population absolute numbers of sequences found in all three replicates were higher, while in naive subsets from both young and elderly these numbers were lower, except for a few cases (Table S7 in Supplementary Material).

To understand whether the recurrence of clonotypes would be associated with particular infections, we next evaluated receptor clonotypes linked to pathogens such as *M. tuberculosis* (16, 47) and herpes viruses such as CMV (18). Whereas in our healthy controls no *M. tuberculosis*-specific clonotypes could be identified, CMV-specific TRG or TRD clonotypes were found in most controls, and in one case even a complete CMV-specific TCRγδ receptor could be identified (data not shown). However, there were no evident differences between young and elderly individuals.

Finally, as leukemic TCRγδ+ T-cells typically arise in the elderly population and are associated with specific clonotypes, we retrospectively reviewed our TCRγδ+ T-cell LGL leukemia database of clonal TRG/TRD sequences (13, 47) and searched for these LGL clonotypes in the normal TCRγδ+ T-cell repertoire of young and elderly healthy individuals. Interestingly, two TCRγδ+ T-LGL leukemia-associated TRG and TRD clonotypes were found in four older individuals and in one young individual (Table 1). The Vδ3–Jδ1 receptor as identified in TCRγδ+ T-LGL leukemia case 12-098 was identified in one young individual (naive subset, 26-year-old female), and in three older individuals (naive subset, 56-year-old male; effector subset, 69-year-old female and 68-year-old male) (Table 1). The TCRγδ+ T-LGL leukemia-related receptor from case 10 to 200 was found twice in older individuals (naive subset, 56-year-old male; effector subset, 70-year-old male) (Table 1). Although the numbers are low, the fact that two TCRγδ+ T-LGL leukemia-related receptors could specifically be identified in effector cells of elderly would support the idea that TCRγδ+ T-LGL leukemia cells originate from the normal TCRγδ repertoire, especially from antigen-experienced TCRγδ+ T-cells of individuals of older age (13, 48, 49).

**Table 1:** Complete TCRγδ+ T-LGL leukemia receptor clonotypes identified in the repertoire of healthy young and elderly individuals.

Sample information				LGL sample	TRG receptor chain		TRD receptor chain	
Donor	Sex	Age	Subset		V-J rearrangement	CDR3 composition	V-J rearrangement	CDR3 composition
**Young individuals**								
B49	F	26	Naive	LGL 12-098	Vδ3–Jδ1	CAFSSLTGGYKEYTDKLIF	Vγ9–Jγ1.3	CALWEVPNYKKLF

**Elderly individuals**								
B41	M	56	Naive	LGL 10-200	Vδ2–Jδ1	CACDTVGDRDTDKLIF	Vγ9–Jγ1.3	CALWEVQYYKKLF
				LGL 12-098	Vδ3–Jδ1	CAFSSLTGGYKEYTDKLIF	Vγ9–Jγ1.3	CALWEVPNYKKLF
B51	M	70	Effector	LGL 10-200	Vδ2–Jδ1	CACDTVGDRDTDKLIF	Vγ9–Jγ1.3	CALWEVQYYKKLF
B44	F	69	Effector	LGL 12-098	Vδ3–Jδ1	CAFSSLTGGYKEYTDKLIF	Vγ9–Jγ1.3	CALWEVPNYKKLF
B60	M	68	Effector	LGL 12-098	Vδ3–Jδ1	CAFSSLTGGYKEYTDKLIF	Vγ9–Jγ1.3	CALWEVPNYKKLF

## Discussion

Aging of the immune system has become increasingly important due to increased hygiene and higher life expectancies in the Western World (3, 5, 50). Immunosenescence plays an additional role in shaping the immune repertoire. Shaping of the immune system during ontogeny and upon aging relies on continuous antigenic exposures, varying from pathogens to cellular stress. In the current study, we showed that (antigenic) selection starts during early ontogeny in the thymus and CB samples and continues in circulating TCRγδ+ T-cells in young and elderly individuals. While maintaining diversity in the naive subsets, the effect of aging is most significant in memory subsets, characterized by strong receptor skewing, and in effector subsets.

Following technical optimization of multiplex PCR assays for NGS analysis, we demonstrated highly diverse TRG, but Vδ1-skewed TCRγδ+ T-cell repertoires in precursor TCRγδ+ T-cells from thymus and CB, with low inter-sample variation. This was in clear contrast to circulating mature TCRγδ+ T-cells that showed Vγ9/Vδ2 receptor skewing with high inter-sample variation and donor-specific patterns. As we recently showed significant effects of aging on maturation profiles of TCRγδ+ T-cells (49), we investigated the immune repertoire composition of different TCRγδ+ T-cell subsets including naive, central, and effector memory, and effector cells. Even though the naive TCRγδ+ T-cell population shrinks upon aging (3, 5, 49), its diversity—being the primary source for mounting immune responses—was maintained in elderly individuals. To date, only one study documented the maintenance of the naive CD4+ TCRαβ+ T-cell repertoire until the age of 70, after which the repertoire profoundly declined (51) [reviewed in Ref. (52)]. These results are in line with our findings, although our cohort consisted of elderly until the age of 70. This is one drawback of our study, but the maximum age to donate blood at our national blood bank is 70. Nevertheless, it would be interesting to also study healthy individuals >70 years of age, although the high volumes of blood needed to obtain sufficient numbers of naiveTCRγδ+ T-cells could complicate such studies. Low cell numbers pose serious limitations to studying the repertoire due to potentially low levels of input DNA and skewed data. To overcome such limitations and to directly link overall receptor usage for both TRG and TRD loci, single molecule-based assays could be considered. However, these assays are rather novel and require extensive optimization and validation experiments as well. Another limitation of our study is the fact that due to limited cell material, we could not go into mechanistic and functional implications of our findings.

Age-related differences were most evident in central and effector memory populations: Vγ9 usage was highly important in young individuals, while a shift toward Vγ2 and other Vγ1-family genes was observed in effector memory TCRγδ+ T-cells of elderly. The significant increase in Vγ2 usage in elderly was accompanied by a significant decrease in Vδ2 and increase in Vδ1 usage, collectively indicating a shift from Vγ9/Vδ2 specificity in young to Vγ2/Vδ1 in elderly. These findings might suggest differences in antigenic selection, or might be due to underlying clonal expansion in these populations (53). Also, CMV is known to elicit Vδ1+ TCRγδ+ T-cell-specific responses (17). Additionally, we have recently demonstrated the effect of CMV on the TCRγδ+ T-cell immune system, through increasing Vδ1+ TCRγδ+ T-cells in elderly carrying CMV (49), and it has been shown that latent CMV carriage is related to the expansion of CMV specific T-cells (54). When zooming in on the antigen-binding part, the CDR3 region, we could indeed identify CMV-specific CDR3 regions in a few donors, albeit without a significant aging effect. This could reflect high anti-CMV responses mounted by Vδ1+ TCRγδ+ T-cells, although such responses were mainly observed in renal allograft recipients and not in healthy controls (17). Given that CMV infects mainly fibroblasts and epithelial cells (55), and that the majority of Vδ1+ TCRγδ+ T-cells reside in epithelial and mucosal tissues (56–58) these findings could indicate that healthy individuals have a local, rather than circulatory, protection by Vδ1+ TCRγδ+ T-cells against CMV. Interestingly, in a recent study Davey et al., showed that the Vδ1 population in CB is unfocused, but that in adult PB clonal expansions could be found that had directly differentiated from naive into effector phenotypes with parallel CD27 downregulation (59). In contrast, Vδ2 cells maintained their TCR expression from birth to adulthood. Together with the CMV effect upon aging, these findings could explain the higher Vδ1 usage in elderly and possibly the occurrence of clonopathies.

In this study, we also examined other dominant TRG/TRD clonotypes, such as for *M. tuberculosis*, since TCRγδ+ T-cells are known to elicit strong responses (16), but these were not identified. This could be related to the recruitment of our donors (mostly of Caucasian descent) *via* the national Dutch blood bank, and the fact that donors are tested prior to blood donation. Furthermore, open tuberculosis is not endemic in the Netherlands. Curiously, some TRG/TRD clonotypes derived from complete TCRγδ+ T-LGL leukemia receptors were identified in the healthy effector subset repertoire. These findings would be in line with earlier correlations identified between TCRγδ+ T-LGL leukemia cells and healthy effector TCRγδ+ T-cells (49) and would support the concept that TCRγδ+ T-LGL is a disease that typically arises in effector cells of elderly.

Our optimized multiplex PCR assays for NGS analysis could also be applicable to other disease states, such as TCRγδ+ T-cell lymphomas, or treatments, such as bone marrow transplantation (BMTx). TCRγδ+ T-cells have been described to reconstitute in increased numbers after BMTx in acute leukemia patients (60). The here described method would allow to investigate to what extent the TRG/TRD repertoire has changed upon BMTx, and how the TCRγδ+ T-cell compartment regenerates. Also, circulating TCRγδ+ T-cells have been described in metastatic melanomas, in which it would be interesting to distinguish pro- and anti-tumor specific TCRγδ+ T-cells (61), the latter particularly in view of tumor-eradicating effects (45, 62). Also, investigating the TRG/TRD repertoires of tissue-residing TCRγδ+ T-cells could be relevant, not only for CMV-specific responses, but also for other local antigens contributing to the TCRγδ+ T-cell repertoire. As the ability of TCRγδ+ T-cells to move in and out of tissues has not been convincingly demonstrated yet, sequencing TCRγδ+ T-cells from different tissues could provide insight in both residing, migrating and circulating properties, as well as in development of local immune repertoires and additional functions and specificities of TCRγδ+ T-cells.

In summary, using an optimized NGS assay we identified specific TRG/TRD repertoires during ontogeny and upon aging. Despite strong individual-specific repertoire compositions, significant differences in Vγ and Vδ gene usage were identified upon aging in especially the memory TCRγδ+ T-cell subsets. These age-dependent effects caused shifts from Vγ9/Vδ2 dominance in young to Vγ2/Vδ1 dominance in elderly. Additionally, some TRG/TRD clonotypes related to TCRγδ+ T-LGL leukemia were identified in normal effector TCRγδ+ T-cells of especially elderly individuals, which fits the idea that TCRγδ+ T-LGL leukemia originates from normal circulating, antigen-experienced effector TCRγδ+ T-cells.

## Ethics Statement

Blood from healthy blood donors from Sanquin Blood Bank (Amsterdam, The Netherlands) in the age ranges 20–35 (young adults) and 56–70 (elderly) was used upon informed consent (project number NVT0012.01) and anonymized for further use. Healthy neonatal CB was obtained postpartum or after Caesarian section upon informed consent through collaboration with the departments of Obstetrics and Hematology. Thymic lobes were removed upon heart surgery in individuals under the age of 2 years. Both CB and thymus material was obtained under Medical Ethics Committee approval (project number hmPOO2004-003). All studies were conducted in accordance with the principles of the Declaration of Helsinki.

## Author Contributions

MJK, JD, and AL designed the experiments. MJK and AL wrote the manuscript. MJK, FK, MYK, and IW-T performed the experiments. MJK and AL analyzed the data and prepared the figures. PV, JD, and AL supervised the project. FK and PV revised the manuscript. All authors read the manuscript carefully.

## Conflict of Interest Statement

The authors declare that the research was conducted in the absence of any commercial or financial relationships that could be construed as a potential conflict of interest.

## References

[1] Wherry EJ T-cell exhaustion. Nat Immunol (2011) 12(6):492–9. 10.1038/ni.2035 21739672

[2] Wherry EJ Kurachi M Molecular and cellular insights into T-cell exhaustion. Nat Rev Immunol (2015) 15(8):486–99. 10.1038/nri/3862 26205583 PMC4889009

[3] Boraschi D Aguado MT Dutel C Goronzy J Louis J Grubeck-Loebenstein B The gracefully aging immune system. Sci Transl Med (2013) 5(185):185s8 10.1126/scitranslmed.3005624 23677590

[4] Aspinall R Andrew P Thymic involution in aging. J Clin Immunol (2000) 20(4):250–6. 10.1023/A:1006611518223 10939712

[5] Weiskopf D Weinberger B Grubeck-Loebenstein B. The aging of the immune system. Transpl Int (2009) 22:1041–50. 10.1111/j.1432-2277.2009.00927.x 19624493

[6] Pereira BI Akbar AN. Convergence of innate and adaptive immunity during human aging. Front Immunol (2016) 7:445. 10.3389/fimmu.2016.00445 27867379 PMC5095488

[7] Oettinger MA Schatz DG Gorka C Baltimore D. RAG-1 and RAG-2 adjacent genes that synergistically activate V(D)J recombination. Science (1990) 248(4962):1517–23. 10.1126/science.2360047 2360047

[8] Dik WA Pike-Overzet K Weerkamp F de Ridder D de Haas EF Baert MR New insights on human T-cell development by quantitative T-cell receptor gene rearrangement studies and gene expression profiling. J Exp Med (2005) 201(11):1715–23. 10.1084/jem.20042524 15928199 PMC2213269

[9] Fu G Rybakin V Brzostek J Paster W Acuto O Gascoigne NRJ Fine-tuning T-cell receptor signaling to control T-cell development. Trends Immunol (2014) 35(7):311–8. 10.1016/j.it.2014.05.003 24951034 PMC4119814

[10] Van Dongen JJM Comans-Bitter WM Wolvers-Tettero ILM Borst J Development of human T lymphocytes and their thymus-dependency. Thymus (1990) 16(3–4):207–34. 2293424

[11] Parker CM Groh V Band H Porcelli SA Morita C Fabbi M Evidence for extrathymic changes in the T-cell receptor gamma/delta repertoire. J Exp Med (1990) 171(5):1597–612. 10.1084/jem.171.5.1597 2185330 PMC2187908

[12] Prinz I Silva-Santos B Pennington DJ Functional development of γδ T-cells. Eur J Immunol (2013) 43(8):1988–94. 10.1002/eji.201343759 23928962

[13] Sandberg Y Almeida J Gonzalez M Lima M Bárcena P Szczepanski T TCRγδ+ large granular lymphocyte leukemias reflect the spectrum of normal antigen-selected TCRγδ+ T-cells. Leukemia (2006) 20:505–13. 10.1038/sj.leu.2404112 16437145

[14] Davodeau F Peyrat MA Hallet MM Houde I Vie H Bonneville M Peripheral selection of antigen receptor junctional features in a major human γδ subset. Eur J Immunol (1993) 23:804–8. 10.1002/eji.1830230405 8384559

[15] Breit TM Wolvers-Tettero ILM van Dongen JJM Unique selection determinant in polyclonal Vδ2 – Jδ1 junctional regions of human peripheral γδ T lymphocytes. J Immunol (1994) 152:2860–4. 7511631

[16] Xi X Han X Li L Zhao Z Gammadelta T-cells response to *Mycobacterium tuberculosis* in pulmonary tuberculosis patients using preponderant complementary determinant region 3 sequence. Indian J Med Res (2011) 134:356–61. 21985819 PMC3193717

[17] Déchanet J Merville P Lim A Retière C Pitard V Lafarge X Implication of γδ T-cells in the human immune response to cytomegalovirus. J Clin Invest (1999) 103:1437–49. 10.1172/JCI5409 10330426 PMC408467

[18] Vermijlen D Brouwer M Donner C Liesnard C Tackoen M van Rysselberge M Human cytomegalovirus elicits fetal γδ T-cell responses in utero. J Exp Med (2010) 207(4):807–21. 10.1084/jem.20090348 20368575 PMC2856038

[19] Fujishima N Hirokawa M Fujishima M Yamashita J Saitoh H Ichikawa Y Skewed T-cell receptor repertoire of Vδ1+ γδ T lymphocytes after human allogeneic haematopoietic stem cell transplantation and the potential role for Epstein-Barr virus-infected B cells in clonal restriction. Clin Exp Immunol (2007) 149:70–9. 10.1111/j.1365-2249.2007.03388.x 17425654 PMC1942033

[20] De Jong A Activation of human T-cells by CD1 and self-lipids. Immunol Rev (2015) 267(1):16–29. 10.111/imr.12322 26284469 PMC6673648

[21] Uldrich AP Le Nours J Pellicci DG Gherardin NA McPherson KG Lim RT CD1d-lipid antigen recognition by the γδ TCR. Nat Immunol (2013) 14(11):1137–45. 10.1038/ni.2713 24076636

[22] Kabelitz D Lettau M Janssen O. Immunosurveillance by human γδ T lymphocytes: the emerging role of butyrophilins. F100Res (2017) 6:782. 10.12688/f1000research.11057 28649364 PMC5464295

[23] Vasudev A Ying CTT Ayyadhury S Puan KJ Andiappan AK Nyunt MSZ γ/δ T cell subsets in human aging use the classical α/β T cell model. J Leukoc Biol (2014) 8:647–55. 10.1189/jlb.5A1213-650RR 25001861

[24] Van Dongen JJM Langerak AW Brüggeman M Evans PAS Hummel M Lavender FL Design and standardization of PCR primers and protocols for detection of clonal immunoglobulin and T-cell receptor gene recombinations in suspect lymphoproliferations: report of the BIOMED-2 concerted action BMH4-CT98-3936. Leukemia (2003) 17:2257–317. 10.1038/sj.leu.2403202 14671650

[25] Sandberg Y Verhaaf B van Gastel-Mol EJ Wolvers-Tettero ILM de Vos AW Noordzij JG Human T-cell lines with well-defined T-cell receptor gene rearrangements as controls for the BIOMED-2 multiplex polymerase chain reaction tubes. Leukemia (2007) 21:230–7. 10.1038/sj.leu.2404486 17170727

[26] Moorhouse MJ van Zessen D Ijspeert H Hiltemann S Horsman S van der Spek PJ ImmunoGlobulin galaxy (IGGalaxy) for simple determination and quanti-tation of immunoglobulin heavy chain rearrangements from NGS. BMC Immunol (2014) 15:59. 10.1186/s12865-014-0059-7 25495099 PMC4282729

[27] Goecks J Nekrutenko A Taylor J The Galaxy Team. Galaxy: a comprehensive approach for supporting accessible, reproducible, and transparent computational research in the life sciences. Genome Biol (2010) 11(8):R86. 10.1186/gb-2010-11-8-r86 20738864 PMC2945788

[28] Blankenberg D Von Kuster G Coraor N Ananda G Lazarus R Mangan M Galaxy: a web-based genome analysis tool for experimentalists. Curr Protoc Mol Biol (2010) 19:.1–.21. 10.1002/0471142727.mb1910s89 20069535 PMC4264107

[29] Giardine B Riemer C Hardison RC Burhans R Elnitski L Shah P Galaxy: a platform for interactive large-scale genome analysis. Genome Res (2005) 15(10):1451–5. 10.1101/gr.4086505 16169926 PMC1240089

[30] Blankenberg D Gordon A Von Kuster G Coraor N Taylor J Nekrutenko A Manipulation of FASTQ data with galaxy. Bioinformatics (2010) 26(14):1783–5. 10.1093/bioinformatics/btg281 20562416 PMC2894519

[31] Alamyar E Giudicelli V Shuo L Duroux P Lefranc M-P IMGT/HighV-QUEST: the IMGT^®^ web portal for immunoglobulin (IG) or antibody and T-cell receptor (TR) analysis from NGS high throughput and deep sequencing. Immunome Res (2012) 8(1):26 10.1007/978-1-61779-842-9_32

[32] Alamyar E Duroux P Lefranc MP Giudicelli V IMGT(^®^) tools for the nucleotide analysis of immunoglobulin (IG) and T-cell receptor (TR) V-(D)-J repertoires, polymorphisms, and IG mutations: IMGT/V-QUEST and IMGT/HighV-QUEST for NGS. Methods Mol Biol (2012) 882:569–604. 10.1007/987-1-61779-842-9_32 22665256

[33] Li S Lefranc MP Miles JJ Alamyar E Giudicelli V Duroux P IMGT/HighV QUEST paradigm for T-cell receptor IMGT clonotype diversity and next-generation repertoire immunoprofiling. Nat Commun (2013) 4:2333 10.1038/ncomms3333 23995877 PMC3778833

[34] Giudicelli V Duroux P Lavoie A Aouinti S Lefranc MP Kossida S From IMGT-ONTOLOGY to IMGT/HighVQUEST for NGS Immunoglobulin (IG) and T-cell receptor (TR) repertoires in autoimmune and infectious diseases. Autoimmun Infect Dis (2015) 1(1):1–16. 10.16966/aidoa.103

[35] Boyd SD Marshall EL Merker JD Maniar JM Zhang LN Sahaf B Measurement and clinical monitoring of human lymphocyte clonality by massively parallel VDJ pyrosequencing. Sci Transl Med (2009) 1(12):12ra23. 10.1126/scitranslmed.3000540 20161664 PMC2819115

[36] Krzywinksi MI Schein JE Birol I Connors J Gascoyne R Horsman D Circos: an information aesthetic for comparative genomic. Genome Res (2009) 19:1639–45. 10.1101/gr.092759.109 19541911 PMC2752132

[37] Crooks GE Hon G Chandonia JM Brenner SE. WebLogo: a sequence logo generator. Genome Res (2004) 14:1188–90. 10.1101/gr.849004 15173120 PMC419797

[38] Schneider TD Stephens RM. Sequence logos: a new way to display consensus sequences. Nucleic Acids Res (1990) 18:6097–100. 10.1093/nar/18.20.6097 2172928 PMC332411

[39] Hartigan PM Algorithm AS 217: computation of the dip statistic to test for unimodality. Appl Stat (1985) 34:320–5. 10.2307/2347485

[40] Hartigan JA Hartigan PM The dip test of unimodality. Ann Stat (1985) 13:70–84. 10.1214/aos/1176346577

[41] Mächler M Diptest 0.25-1. (2003). Available from: http://www.r-project.org/

[42] Core Team R R: a language and environment for statistical computing. R Foundation for Statistical Computing. Vienna, Austria (2016). https://www.R-project.org

[43] Liston A Carr EJ Linterman MA Shaping variation in the human immune system. Trends Immunol (2016) 36(10):637–46. 10.1016/j.it.2016.08.002 27692231

[44] Kallemeijn MJ Boots AMH van der Klift MY Brouwer E Abdulahad WH Verhaar JAN Ageing and latent CMV infection impact on maturation, differentiation and exhaustion profiles of T-cell receptor gammadelta T-cells. Sci Rep (2017) 7:5509. 10.1038/s41598-017-05849-1 28710491 PMC5511140

[45] Correia DV d’Orey F Cardoso BA Lança T Grosso AR deBarros A Highly active microbial phosphoantigen induces rapid yet sustained MEK/Erk- and PI-3K/Akt-mediated signal transduction in anti-tumor human gammadelta T-cells. PLoS One (2009) 4(5):e5657. 10.1371/journal.pone.0005657 19479075 PMC2682580

[46] Ijspeert H van Schouwenburg PA van Zessen D Pico-Knijnenburg I Stubbs AP van der Burg M. Antigen receptor galaxy: a user-friendly, web-based tool for analysis and visualization of T and B cell receptor repertoire data. J Immunol (2017) 198:4156–65. 10.4049/jimmunol.1601924 28416602 PMC5421304

[47] Spencer CT Abate G Blazevic A Hoft DF Only a subset of phosphoantigen-responsive gamma9delta2 T-cells mediate protective tuberculosis immunity. J Immunol (2008) 181:4471–84. 10.4049/jimmunol.181.7.4471 18802050 PMC2670066

[48] Sandberg Y Kallemeijn MJ Dik WA Tielemans D Wolvers-Tettero ILM van Gastel-Mol EJ Lack of common TCRA and TCRB clonotypes in CD8(+)/TCRαβ(+) T-cell large granular lymphocyte leukemia: a review on the role of antigenic selection in the immunopathogenesis of CD8(+) T-LGL. Blood Cancer J (2014) 10(4):e172. 10.1038/bcj.2013.70 24413066 PMC3913939

[49] Kallemeijn MJ de Ridder D Schilperoord-Vermeulen J van der Klift MY Sandberg Y van Dongen JJ Dysregulated signaling, proliferation and apoptosis impact on the pathogenesis of TCRγδ+ T-cell large granular lymphocyte leukemia. PLoS One (2017) 12(4):e0175670 10.1371/journal.pone.0175670 28407008 PMC5391076

[50] World Health Organization. World Report on Aging and Health. Luxembourg: WHO (2015).

[51] Naylor K Li G Vallejo AN Lee WW Koetz K Bryl E The influence of age on T cell generation and TCR diversity. J Immunol (2005) 174:7446–52. 10.4049/jimmunol.174.11.7446 15905594

[52] Appay V Sauce D. Naive T cells: the crux of cellular immune aging? Exp Gerontol (2014) 54:90–3. 10.1016/j.exger.2014.01.003 24440387

[53] Yoshida K Cologne JB Cordova K Misumi M Yamoaka M Kyoizumi S Aging-related changes in human T-cell repertoire over 20 years delineated by deep sequencing of peripheral T-cell receptors. Exp Gerontol (2017) 96:29–37. 10.1016/j.exger.2017.05.015 28535950

[54] Jackson SE Sedikides GX Okecha G Poole EL Sinclair JH Wills MR. Latent cytomegalovirus (CMV) infection does not detrimentally alter T cell responses in the healthy old, but increased latent CMV carriage is related to expanded CMV-specific T cells. Front Immunol (2017) 8:733. 10.3389/fimmu.2017.00733 28694811 PMC5483450

[55] Merani S Pawelec G Kuchel GA McElhaney JE. Impact of aging and Cytomegalovirus on immunological response to Influenza vaccination and infection. Front Immunol (2017) 8:784. 10.3389/fimmu.2017.00784 28769922 PMC5512344

[56] Vantourout P Hayday A Six-of-the-best: unique contributions of γδ T-cells to immunology. Nat Rev Immunol (2013) 13(2):88–100. 10.1038/nri3384 23348415 PMC3951794

[57] Chien YH Meyer C Bonneville M γδ T cells: first line of defense and beyond. Annu Rev Immunol (2014) 32:121–55. 10.1146/annurev-immunol-032713-120216 24387714

[58] Nielsen MM Witherden DA Havran WL. γδ T cells in homeostasis and host defence of epithelial barrier tissues. Nat Rev Immunol (2017) 17(12):733–45. 10.1038/nri.2017.101 28920588 PMC5771804

[59] Davey MS Wlicox CR Joyce SP Ladell K Kasatskaya SA McLaren JE Clonal selection in the human Vδ1 T cell repertoire indicates γδ TCR-dependent adaptive immune surveillance. Nat Commun (2017) 8:14760. 10.1038/ncomms14760 28248310 PMC5337994

[60] Godder KT Henslee-Downey PJ Mehta J Park BS Chiang KY Abhyankar S Long term disease-free survival in acute leukemia patients recovering with increased gammadelta T cells after partially mismatched related donor bone marrow transplantation. Bone Marrow Transplant (2007) 39(12):751–7. 10.1038/sj.bmt.1705650 17450185

[61] Wistuba-Hamprecht K Di Benedetto S Schilling B Sucker A Schadendorf D Garbe C Phenotypic characterization and prognostic impact of circulating γδ and αβ T-cells in metastatic malignant melanoma. Int J Cancer (2016) 138(3):698–704. 10.1002/ijc.29818 26383054

[62] Lafont V Sanchez F Laprevotte E Michaud HA Gros L Eliaou JF Plasticity of γδ T cells: impact on the anti-tumor response. Front Immunol (2014) 5:662 10.3389/fimmu.2014.00622 25538706 PMC4259167

