# Supplementary material for: Next-Generation Sequencing Analysis of the Human TCRγδ+ T-Cell Repertoire Reveals Shifts in Vγ- and Vδ-Usage in Memory Populations upon Aging
Source: Front Immunol. 2018 Mar 6;9:448. doi: 10.3389/fimmu.2018.00448 (PMC5845707; doi:10.3389/fimmu.2018.00448)
Supplement: Supplementary file 1 [file data_sheet_1.PDF]

Supplementary Material

Next-generation sequencing analysis of the human TCRγδ+ T-cell repertoire reveals shifts in Vγ- and Vδ-usage in memory populations upon aging

Kallemeijn MJ[1], Kavelaars FG[2], van der Klift MY[1], Wolvers – Tettero ILM[1], Valk PJM[2], van Dongen JJM[1], Langerak AW[1*]

*** Correspondence:** Corresponding Author: a.langerak@erasmusmc.nl

Supplementary Tables

Supplementary Table 1. Antibody panel used for sorting TCRγδ+ T-cell fractions.

	Fluorochrome							
	**BV421**	**PO**	**FITC**	**PE**	**PE-CF594**	**PE-Cy7**	**APC**	**APC-H7**
**Antibody**	**CD27**	**CD45**	**CD45RO**	**CD197**	**CD3**	**TCRγδ**	**TCRαβ**	**CD45RA**
Clone	O323	HI30	UCHL1	3D13	UCHT1	11F2	IP26	HI100
Manufacturer	BioLegend	Invitrogen	DAKO	e-Biosciences	BD Biosciences	BD Biosciences	e-Biosciences	BD Biosciences

Supplementary Table 2. BIOMED-2 primer sequences for next-generation sequencing methods

Primer name	5'-3' sequence*
Vγ1F	**GGTTGTGTTGGAATCAGGAGTCA**
Vγ9	**CGGCACTGTCAGAAAGGAATC**
Jγ1.1/2.1	**AGTTACTATGAGCTTAGTCCCTT**
Jγ1.3/2.3	**GTGTTGTTCCACTGCCAAAGAG**
Jγ1.2	**TAAGCTTTGTTCCGGGACCA**
Vδ1	**ATGCAAAAAGTGGTCGCTATT**
Vδ2	**ATACCGAGAAAAGGACATCTATG**
Vδ3	**TTTGTCTTTTATGGGGATAACAGCA**
Jδ1	**GTTCCACAGTCACACGGGTTC**
Jδ2	**GTTCCACGATGAGTTGTGTTC**
Jδ3	**CTCACGGGGCTCCACGAAGAG**
Jδ4	**TTGTACCTCCAGATAGGTTCC**

*Primer sequence based on the BIOMED-2 protocol, with small adaptations (24).

Supplementary Table 3. Plasmid spike-in sample compositions for primer titration experiments

**TRD plasmid pool**		**TRG plasmid pool**	
**Rearrangement**	**Source***	**Rearrangement**	**Source***
Vδ1 – Jδ1	ARR	Vγ2-Jγ2.1	Karpas299
Vδ1 – Jδ2	Thymus	Vγ2-Jγ1.3	T005
Vδ1 – Jδ3	Thymus	Vγ2-Jγ2.3	T136
Vδ1 – Jδ4	Thymus	Vγ2-Jγ1.3/2.3	T005
Vδ2 – Jδ1	T103	Vγ3-Jγ1.1	T004
Vδ2 – Jδ2	T036	Vγ3-Jγ2.1	T086
Vδ2 – Jδ3	T142	Vγ3-Jγ1.3	Thymus
Vδ2 – Jδ4	Thymus	Vγ4-Jγ2.1	T095
Vδ3 – Jδ1	T106	Vγ4-Jγ1.3	T068
Vδ3 – Jδ2	T006	Vγ5-Jγ1.3	T018
Vδ3 – Jδ3	Thymus	Vγ8-Jγ1.1	Hut78
Vδ3 – Jδ4	Thymus	Vγ8-Jγ2.1	Molt16
		Vγ8-Jγ1.3	T109
		Vγ8-Jγ2.3	T001
		Vγ9-Jγ1.1	ARR
		Vγ9-Jγ2.1	T013
		Vγ9-Jγ1.2	T167
		Vγ9-Jγ1.3	T018
		Vγ9-Jγ2.3	T106

*Rearrangements were obtained from cell lines (ARR, Molt16, Hut78) (13), thymus material or primary T-ALL samples (T).

Supplementary Table 4. Overview of PCR assay fine-tuning experiments

Variables	EXP2	EXP3				EXP4		EXP5	EXP6		EXP7/8
**Material used***											
	TRG 1 and TRD plasmid pools	TRG 2 and TRD plasmid pools				TRG 2 and TRD plasmid pools		gDNA TH/CB/PB	TRG 2 and TRD plasmid pools		gDNA TH/CB/PB
**Primers (pmol / µl)**											
TRG primer mix		G1				G1	G2	G2	G3	G4	G2
TRGV1F	5	5				5	6	6	6.5	7	6
TRGV9	5	5				5	4	4	3.5	3	4
TRGJP1/P2	3.3	4				4	4	4	4.5	5	4
TRGJ1/J2	3.3	2				2	2	2	1.5	1	2
TRGJP	3.3	4				4	4	4	4	4	4
TRD primer mix		D1	D2	D3	D4	D5	D6	D5	D7	D8	D7
TRDV1	3.3	2	2	2	2	3	3	3	2.5	2	2.5
TRDV2	3.3	6	5	6	5	4	4	4	4.5	5	4.5
TRDV3	3.3	2	3	2	3	3	3	3	3	3	3
TRDJ1	2.5	1.25	1	1	1.25	2	2.5	2	2	2	2
TRDJ2	2.5	3.75	4	4	3.75	2.5	2.5	2.5	2	2	2
TRDJ3	2.5	2.5	2.5	2.5	2.5	2.5	2.5	2.5	2.5	2.5	2.5
TRDJ4	2.5	2.5	2.5	2.5	2.5	2.5	2.5	2.5	2.5	2.5	2.5
	**PCR program variables**										
Ta (ºC)	60	58, 60, 62				58, 59, 60		TRG: 58<br>TRD: 59	TRG: 58<br>TRD: 59		TRG: 58<br>TRD: 59
#Cycles	25	20, 25				20, 25		TRG: 25<br>TRD: 20	TRG: 25<br>TRD: 25		TRG: 25<br>TRD: 25

* Used material for PCR assays either consisted of plasmid pools according to composition summarized in Supplementary Table 3, or of genomic DNA isolated from total TCRγδ+ T cells from thymus material (TH), neonatal cord blood (CB) and adult peripheral blood (PB) (all N=5).

Supplementary Table 5. Optimized protocol for multiplex PCR assays in TRG/TRD NGS experiments

TRG		TRD	
**Primer**	**Concentration (pmol/µl)**	**Primer**	**Concentration (pmol/µl)**
Vγ1F	6	Vδ1	2.5
Vγ9	4	Vδ2	4.5
Jγ1.1/2.1	4	Vδ3	3
Jγ1.3/2.3	2	Jδ1	2
Jγ1.2	4	Jδ2	2
		Jδ3	2.5
		Jδ4	2.5
Tm	58.0	Tm	59.0
Cycle number	25	Cycle number	20/25*

*After TRD biological validation with genomic DNA, the cycle number was adjusted from 20 to 25 cycles to obtain sufficient DNA amplicon for sequencing.

Supplementary Table 6. Donor characteristics and cell numbers of sorted subsets

**Donor characteristics**			**Absolute cell numbers of different TCRγδ+ T-cell subsets***				**Frequencies of different TCRγδ+ T-cell subsets****			
**Donor number**	**Sex**	**Age**	**Naive**	**Central memory**	**Effector memory**	**Effector**	**Naive**	**Central memory**	**Effector memory**	**Effector**
Young (age 20-35)										
B49	F	26	28263	72280	31931	5380	0.30	0.10	26.30	73.30
B50	M	34	17483	150390	14431	1393414	0.05	0.20	9.70	90.05
B53	F	31	28569	80054	924065	790997	0.90	0.30	8.60	90.20
B54	M	30	22959	77880	345713	127702	11.30	3.60	7.20	77.90
B55	M	29	26138	6097	9986	22064	9.90	0.70	9.20	80.20
B58	F	21	22633	17276	112810	16535	27.70	8.90	36.80	26.60
B59	M	28	107919	101833	550244	689371	27.00	16.60	45.60	10.80
B63	F	29	35225	2369	507075	438441	0.90	0.50	19.00	79.60
B67	M	28	9622	1765	100966	68890	1.00	1.80	37.00	60.20
B73	F	20	52185	8722	248533	163600	1.30	4.30	44.40	50.00
B74	M	23	43721	301724	136523	435786	1.45	5.35	44.10	49.10
Elderly (age 56-70)										
B41	M	56	9740	5425	57313	172030	2.20	0.60	27.70	69.50
B42	M	61	2670	3490	46100	119277	1.40	0.30	18.40	79.90
B43	F	56	29390	18095	36855	452425	0.20	0.10	69.50	30.20
B44	F	69	2579	6420	28052	315285	0.20	0.90	30.00	68.90
B45	M	58	7023	12282	164705	137987	0.80	3.40	42.90	52.90
B51	M	70	1950	6443	121493	275430	0.30	0.05	11.20	88.45
B52	F	60	1996	6560	56438	40149	3.70	1.50	33.60	61.20
B60	M	68	95780	331290	18342	420358	1.60	2.10	17.10	79.20
B65	M	62	190507	65911	32914	81127	12.10	6.40	33.60	47.90

B68	M	60	3665	8450	31728	261805	0.60	0.10	4.30	95.00
B69	M	56	18059	4259	179862	2003909	4.10	0.01	39.00	56.89
B70	M	67	6400	1080	33077	23905	1.60	1.60	49.60	47.20

*Subset definitions: naive, CD45RA+CD45RO-CD27+CD197+; central memory, CD45RA-CD45RO+CD27+CD197+; effector memory, CD45RA-CD45RO+CD27-CD197- (Temro); effector, CD45RA+CD45RO-CD27-CD197- (Temra).

**Frequencies of naive, central memory, effector memory and effector cells within total TCRγδ+ T-cell populations.

Supplementary Table 7. Clonality scores and number of coincidences

Sample	Naive*		Effector*		Effector Memory*	
	**Clonality score**	**Coincidence #3****	**Clonality score**	**Coincidence #3****	**Clonality score**	**Coincidence #3****
TRD – Young individuals						
B49	1.25e-06	24	8.98e-05	3	1.58e-04	156
B50	1.30e-04	594	3.45e04	66	2.14e-04	260
B53	9.63e-05	1	3.32e-04	361	1.59e-04	356
B54	3.26e-05	36	2.09e04	454	1.04e-04	339
B55	3.33e-05	1	1.08e04	646	1.63e-04	100
B58	3.46e-04	41	2.22e-05	2	7.74e-05	491
B59	3.32e-05	1	6.42e-05	52	1.10e-04	919
B63	2.13e-04	729	1.53e-04	573	1.15e-04	390
B67	9.57e-05	3353	2.84e-04	617	1.38e-04	342
B73	5.50e-04	15	6.55e-04	81	6.80e-04	165
B74	3.72e-05	20	2.67e-04	342	1.19e-04	441
TRD – Elderly individuals						
B41	1.08e-05	21	5.95e-05	6	4.60e-03	6
B42	3.47e-04	43	1.71e-04	304	4.19e-04	173
B43	1.79e-04	181	1.11e-03	169	3.86e-03	5
B44	7.09e-05	1378	1.40e-03	119	1.84e-04	166
B45	2.49e-04	116	9.21e-04	126	1.15e-04	456
B51	3.12e-04	146	9.33e-04	274	2.68e-04	318
B52	1.80e-04	345	3.94e-04	416	3.48e-04	201
B60	1.39e-04	14	1.14e-03	143	8.19e-04	44
B65	2.01e-04	1122	4.03e-04	355	2.81e-04	66
B68	1.41e-04	12	6.43e-04	182	4.90e-04	73

B69	6.21e-04	172	5.62e-04	154	2.84e-04	73
B70	1.59e-04	59	1.71e-04	528	6.42e-04	247
TRG – Young individuals						
B49	2.00e-04	15	4.22e-05	16	2.49e-05	172
B50	9.37e-05	37	2.22e-04	433	8.27e-04	107
B53	2.81e-05	2	3.07e-04	261	1.06e-03	86
B54	6.33e-05	1888	1.81e-04	407	2.89e-04	295
B55	4.71e-05	34	9.64e-05	79	6.03e-04	35
B58	6.33e-05	464	6.40e-05	13	1.70e-04	281
B59	1.46e-05	10	1.014e-04	35	2.04e-04	595
B63	2.47e-04	777	1.81e-04	31	2.65e-04	253
B67	2.23e-04	10	2.51e-04	667	2.93e-04	259
B73	1.23e-04	152	3.00e-04	83	4.46e-04	221
B74	5.13e-01	402	2.30e-04	227	2.42e-04	295
TRG – Elderly individuals						
B41	2.34e-04	24	3.41e-04	378	2.32e-03	20
B42	1.54e-04	45	3.87e-04	208	2.25e-04	514
B43	2.27e-04	61	3.44e-04	160	5.57e-03	13
B44	1.61e-04	11	1.85e-04	112	9.78e-04	232
B45	1.36e-04	38	2.39e-04	368	1.26e-04	629
B51	2.76e-04	270	1.50e-03	190	2.11e-04	532
B52	2.23e-04	459	3.98e-04	1	2.50e-04	356
B60	7.14e-04	110	8.33e-04	57	5.70e-04	198
B65	3.91e-04	461	1.52e-04	29	3.11e-04	108
B68	1.57e-04	68	5.81e-04	189	3.59e-04	243
B69	1.76e-04	701	1.60e-04	823	3.49e-04	301

B70	2.66e-04	610	2.90e-04	719	6.72e-04	62

*From naive, effector and effector memory subsets 3 replicates were performed; central memory subset was sequenced once due to low DNA yields.

**Absolute number of sequences identified in all three replicates.

Calculations based on the algorithm of Boyd et al. (35).

Supplementary Figures

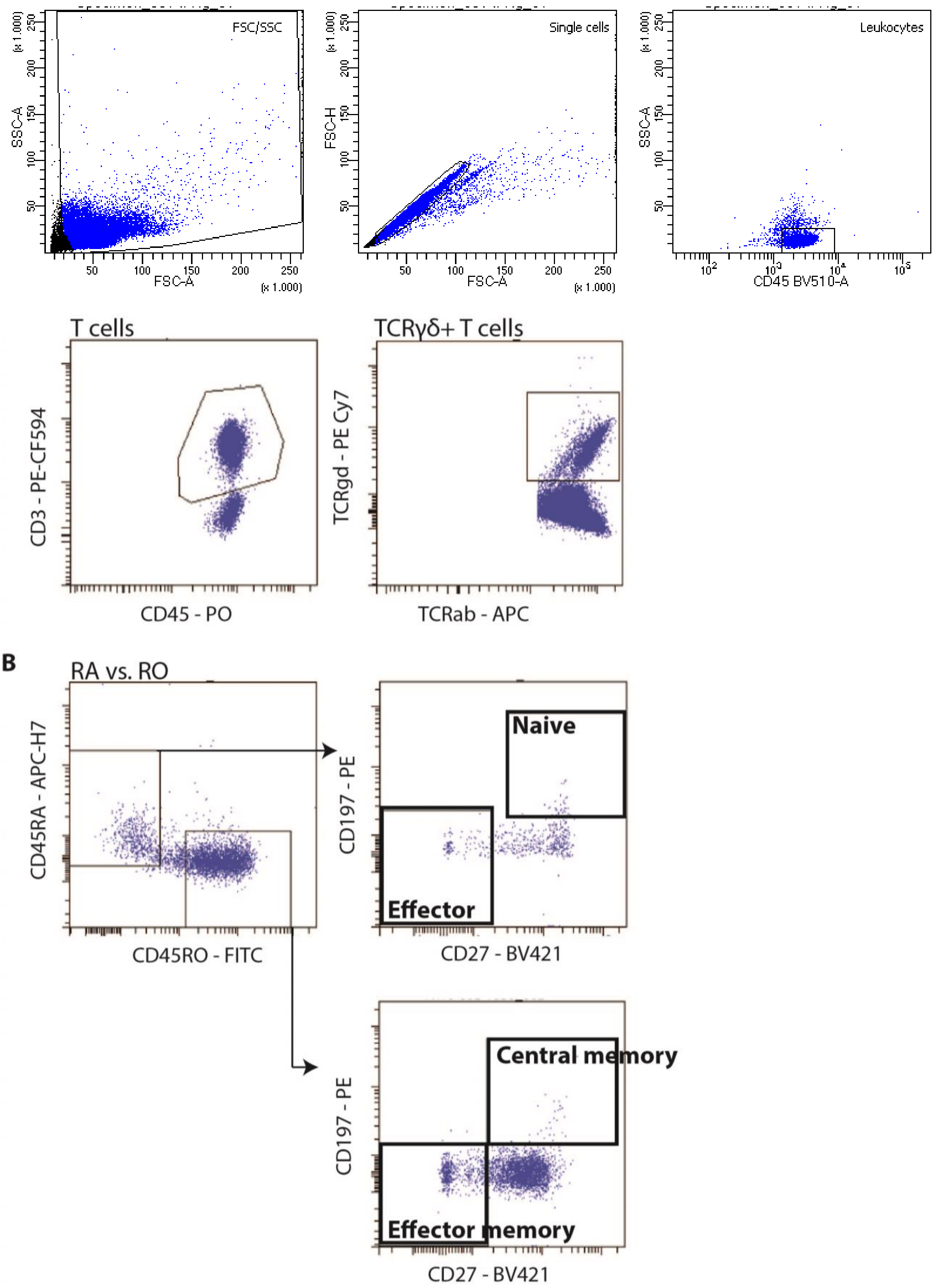

FSC/SSC
SSC-A
FSC-A
Single cells
FSC-H
Leukocytes
CD45 BV510-A
T cells
CD3 - PE-CF594
CD45 - PO
TCRγδ+ T cells
TCRgd - PE Cy7
TCRab - APC
B
RA vs. RO
CD45RA - APC-H7
CD45RO - FITC
Naive
Effector
CD197 - PE
CD27 - BV421
Central memory
Effector memory
CD197 - PE
CD27 - BV421

Supplementary Figure 1. Gating strategies for sorting experiments of TCRγδ+ T-cell subsets. General gating strategies for determining viable cells using FSC/SSC, single cells using FSC-H/FSC-A, leukocytes using CD45, T-cells (CD3+/CD45+) and TCRγδ+ T-cells (TCRγδ+TCRαβ-) **(A)**. Further determination of CD45 splice variants CD45RA vs. CD45RO to sort within CD45RA+CD45RO- for naive (CD27+CD197+) and effector (CD27-CD197-) TCRγδ+ T-cells, and within CD45RA-CD45RO+ for central memory (CD27+CD197+) and effector memory (CD27-CD197-) TCRγδ+ T-cells **(B)**. Antibodies used for these experiments are summarized in Supplementary Table 1.

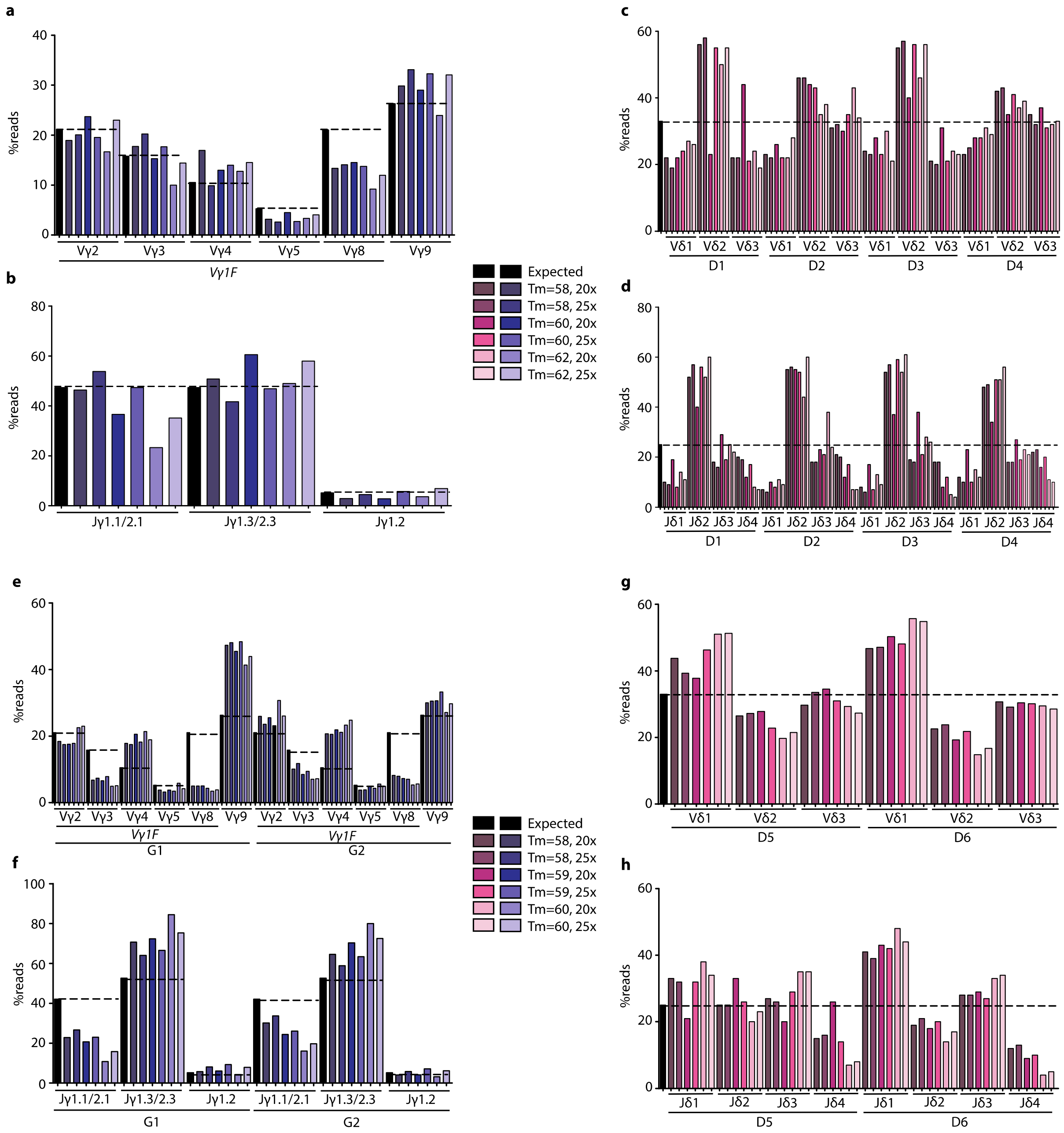

Supplementary Figure 2. TRG / TRD primer titration and PCR optimization experiments. Multiplex PCR tests on plasmid pools with compositions as indicated in Supplementary Table 2 and primer concentrations and PCR assay layout as described in Table 2. Different annealing temperatures (Ta) of 58, 60 and 62 ºa (**A-D**) and 58, 59 and 60 ºC (**E-H**), and two different cycle numbers (20x versus 25x) were tested. The different annealing temperatures and cycle numbers are indicated in the legend with different colors. %Expected indicated with black bar graph and dashed line in graphs. Varying %Expected values for TRGV1F genes due to differential plasmid pool compositions. Each mix, in combination with Ta and cycle number, tested once.

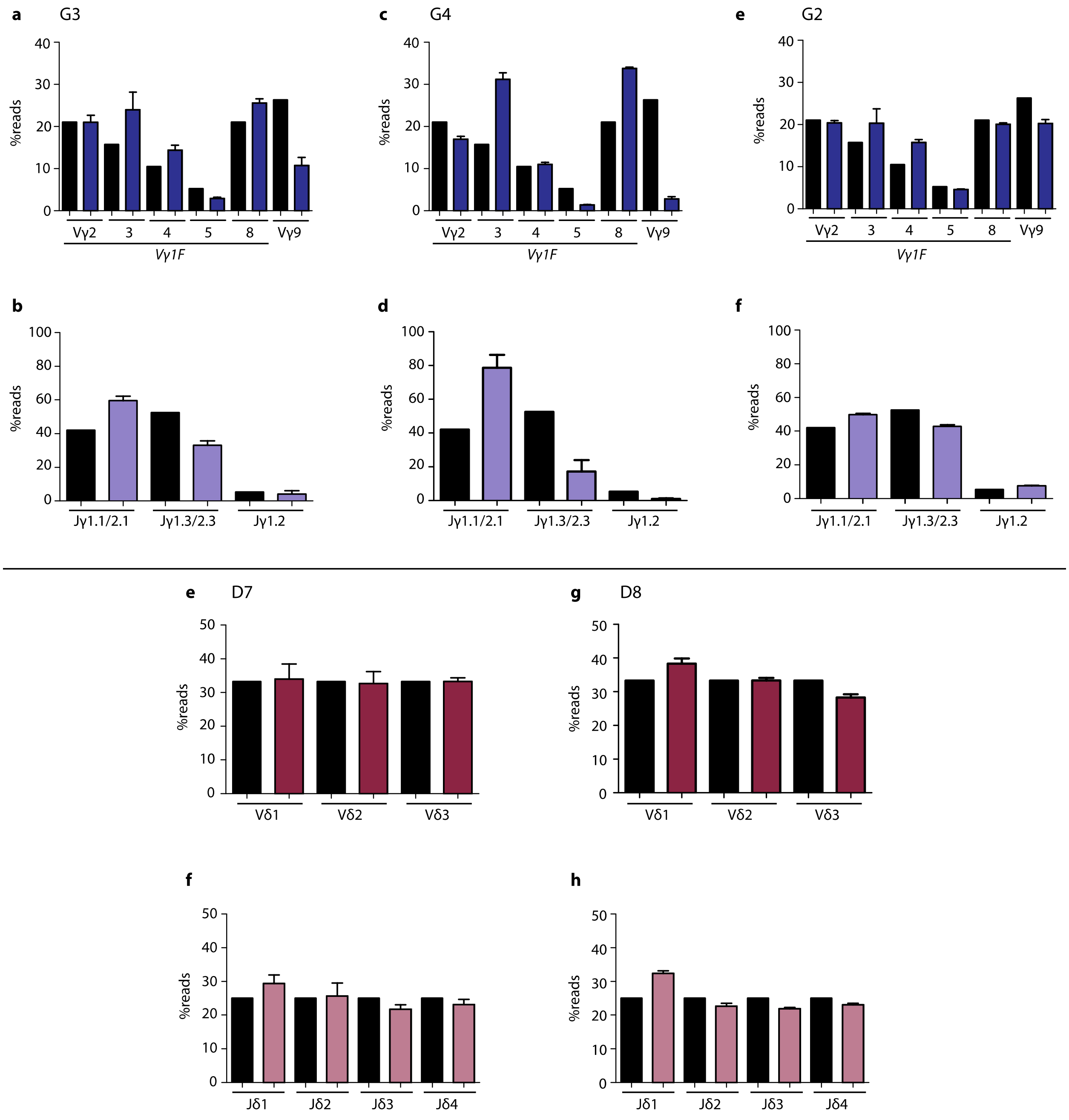

Supplementary Figure 3. Final PCR fine-tuning experiments on plasmid pool DNA leading to most optimal assays. Further attempts for PCR assay optimization to maximally reduce primer bias using multiplex PCR on plasmid pools with compositions indicated in Supplementary Table 3, and primer concentrations and assay numbers as summarized in Supplementary Table 4 with most optimal conditions summarized in Supplementary Table 5. TRGV results (**A,C,E**) and TRGJ results (**B,D,F**) of mixes G3 (**A,B**), G4 (**C,D**) and G2 as comparison (**E,F**). TRDV results (**E,G**) and TRDJ results (**F,H**) of mixes D7 (**E,F**) and D8 (**G,H**).Ta for TRG was set at 58.0, for TRD 59.0ºC. Cycle number for both TRG and TRD PCR assays was 25. Black bars indicate %Expected, colored bars represent the %Observed values. Number of replicates per PCR assay and sample is 4

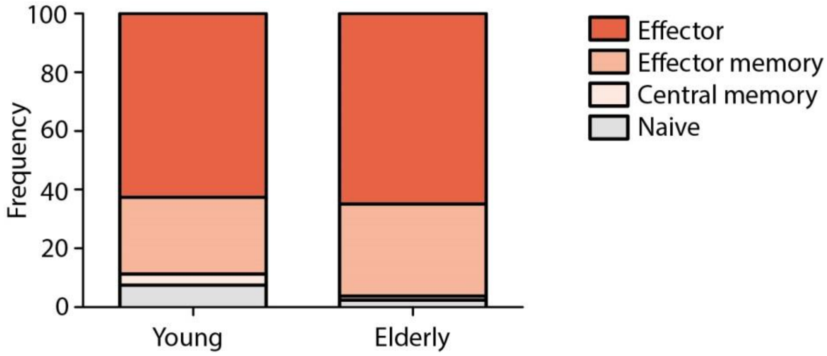

Supplementary Figure 4. Relative TCRγδ+ T-cell subset distributions in young and elderly individuals. Mean frequencies of naive (CD45RA+CD45RO-CD27+CD197+), central memory (CD45RA-CD45RO+CD27+CD197+), effector memory (CD45RA-CD45RO+CD27-CD197-) and effector (CD45RA+CD45RO-CD27-CD197-) TCRγδ+ T-cells as analyzed during sorting experiments. Absolute cell numbers and frequencies are summarized per donor in Supplementary Table 6.

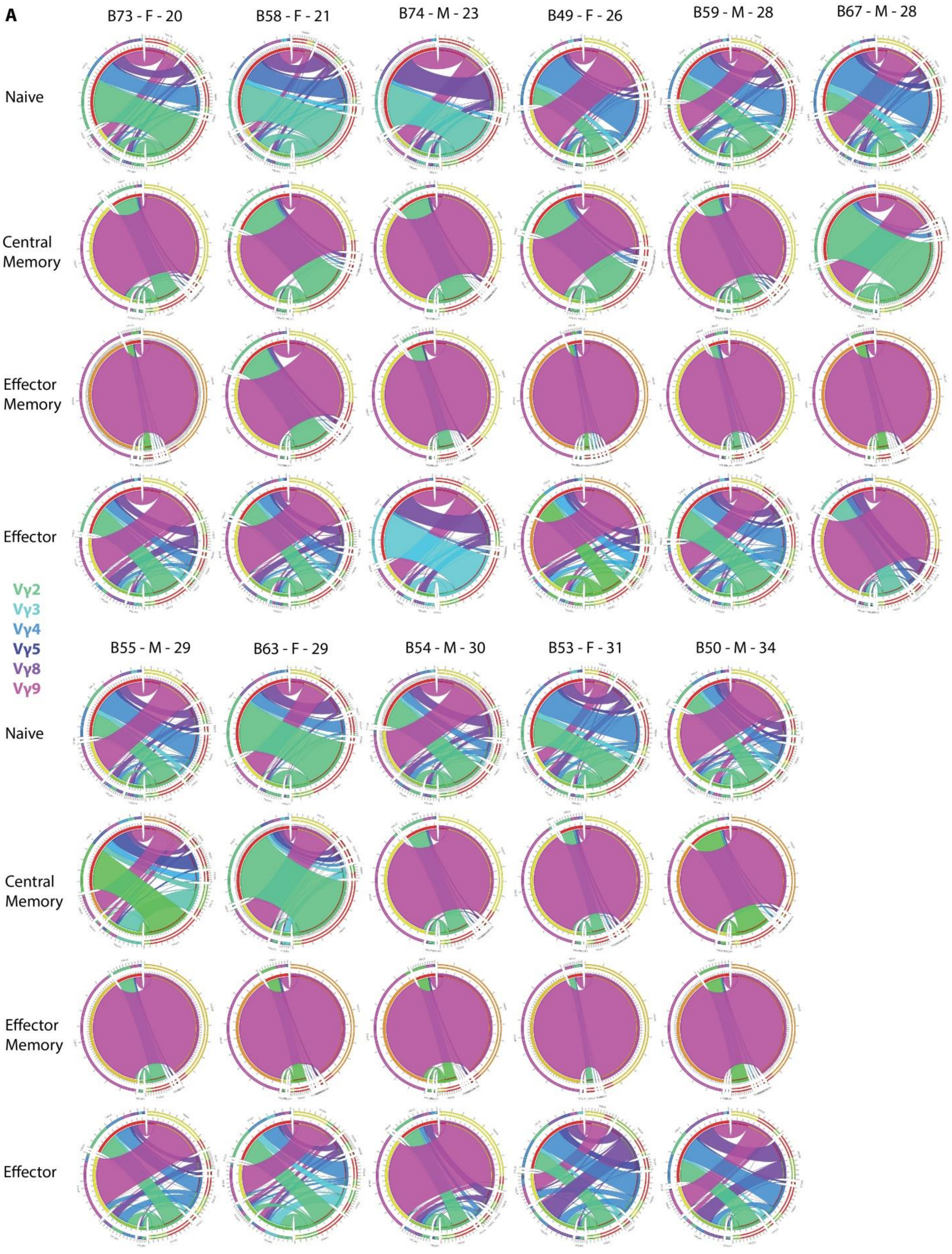
A
B73 - F - 20
B58 - F - 21
B74 - M - 23
B49 - F - 26
B59 - M - 28
B67 - M - 28
Naive
Central Memory
Effector Memory
Effector
Vγ2
Vγ3
Vγ4
Vγ5
Vγ8
Vγ9
B55 - M - 29
B63 - F - 29
B54 - M - 30
B53 - F - 31
B50 - M - 34
Naive
Central Memory
Effector Memory
Effector

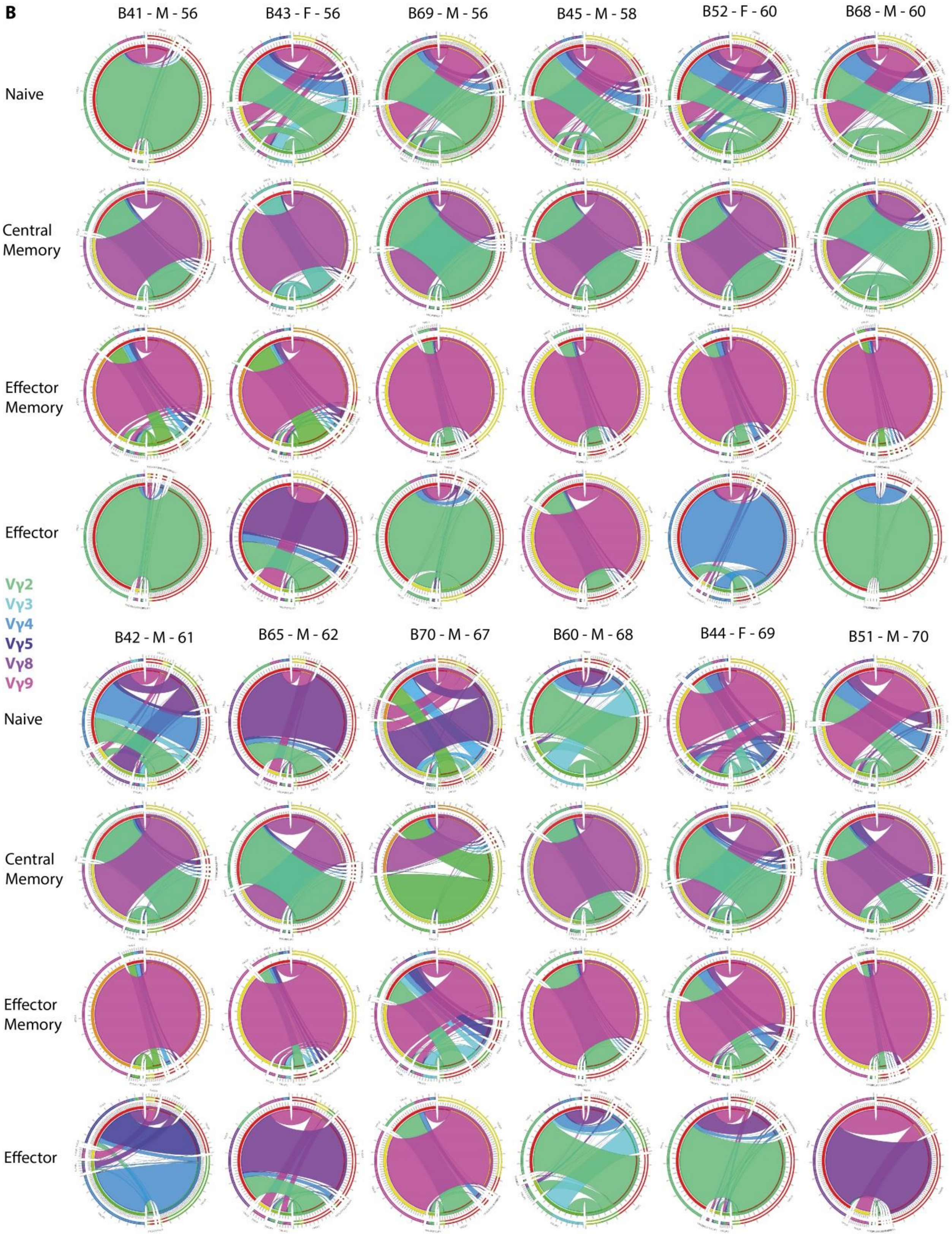

B
B41 - M - 56
B43 - F - 56
B69 - M - 56
B45 - M - 58
B52 - F - 60
B68 - M - 60
Naive
Central Memory
Effector Memory
Effector
Vγ2
Vγ3
Vγ4
Vγ5
Vγ8
Vγ9
B42 - M - 61
B65 - M - 62
B70 - M - 67
B60 - M - 68
B44 - F - 69
B51 - M - 70
Naive
Central Memory
Effector Memory
Effector

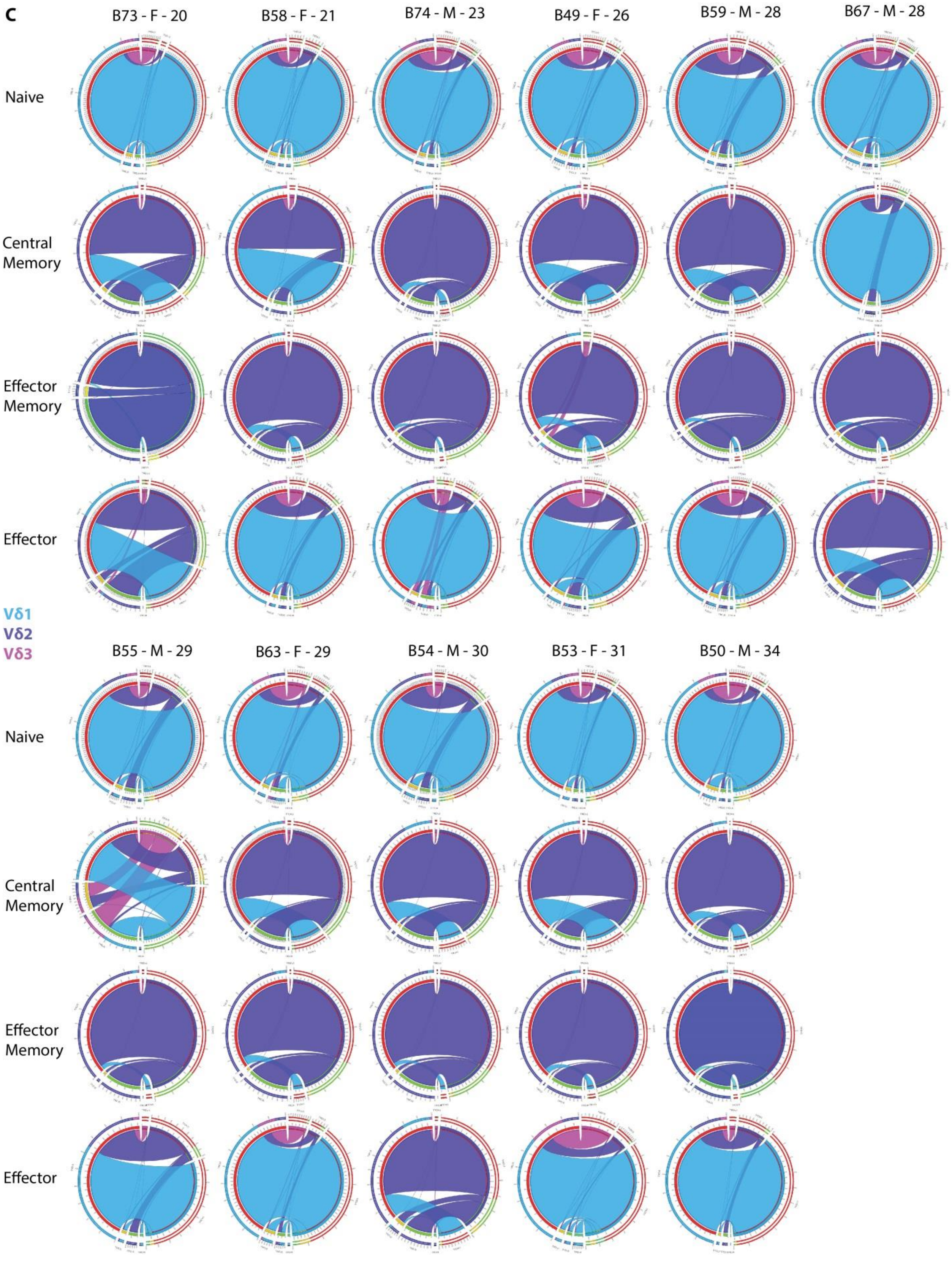
C
B73 - F - 20
B58 - F - 21
B74 - M - 23
B49 - F - 26
B59 - M - 28
B67 - M - 28
Naive
Central Memory
Effector Memory
Effector
Vδ1
Vδ2
Vδ3
B55 - M - 29
B63 - F - 29
B54 - M - 30
B53 - F - 31
B50 - M - 34
Naive
Central Memory
Effector Memory
Effector

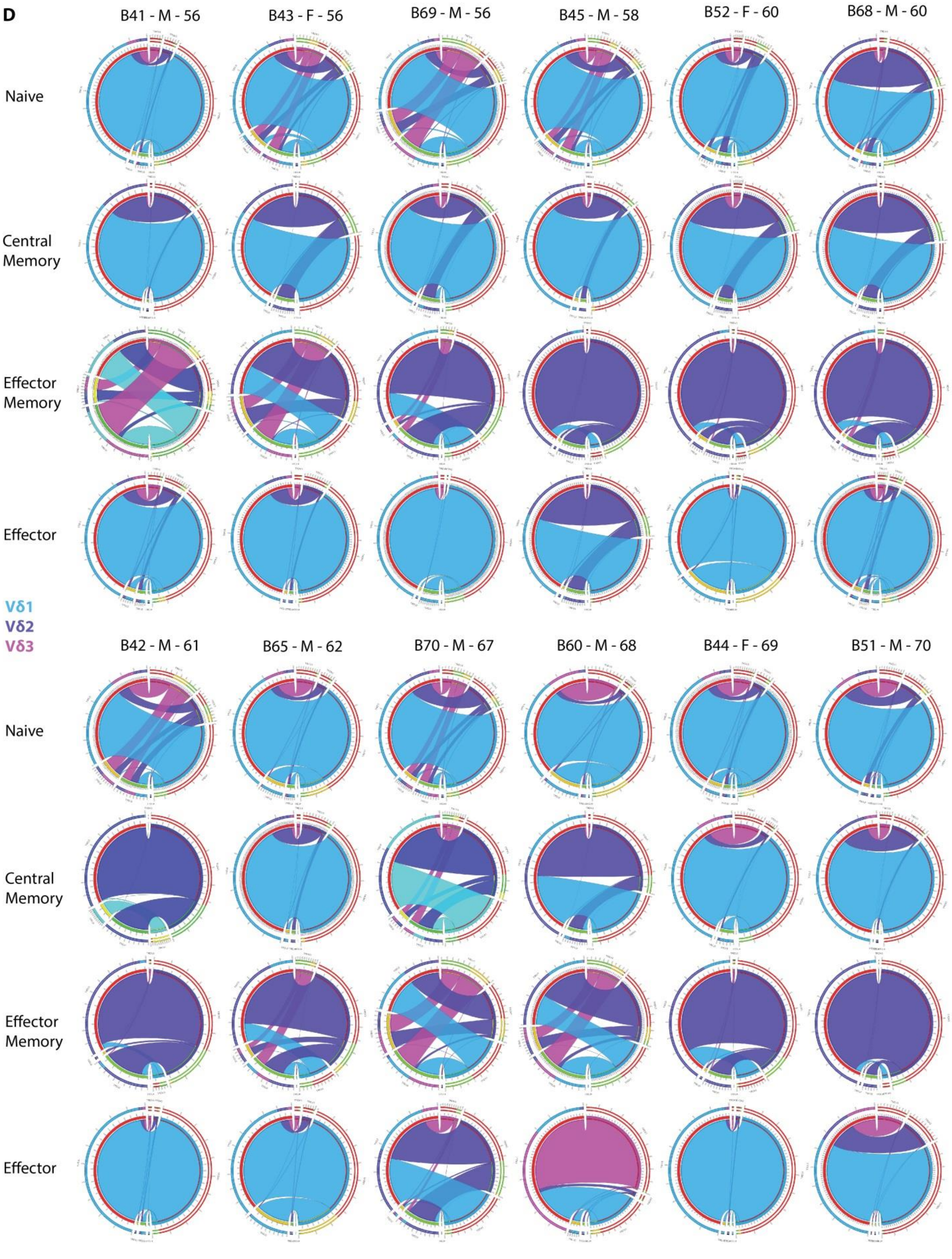
D
B41 - M - 56
B43 - F - 56
B69 - M - 56
B45 - M - 58
B52 - F - 60
B68 - M - 60
Naive
Central Memory
Effector Memory
Effector
Vδ1
Vδ2
Vδ3
B42 - M - 61
B65 - M - 62
B70 - M - 67
B60 - M - 68
B44 - F - 69
B51 - M - 70
Naive
Central Memory
Effector Memory
Effector

Supplementary Figure 5. Circoletto visualizations of naive, central memory, effector memory and effector subsets of young and elderly individuals. V-J distributions of TRG young **(A)**, TRG elderly **(B)**, and TRD young **(C)** and TRD elderly **(D)** rearrangements per donor and subset of young and elderly individuals. Figures were made using the Circoletto online software tool (www.circos.ca) (36)). Each band represents a V-J rearrangement, with different colors reflecting different V gene usage.

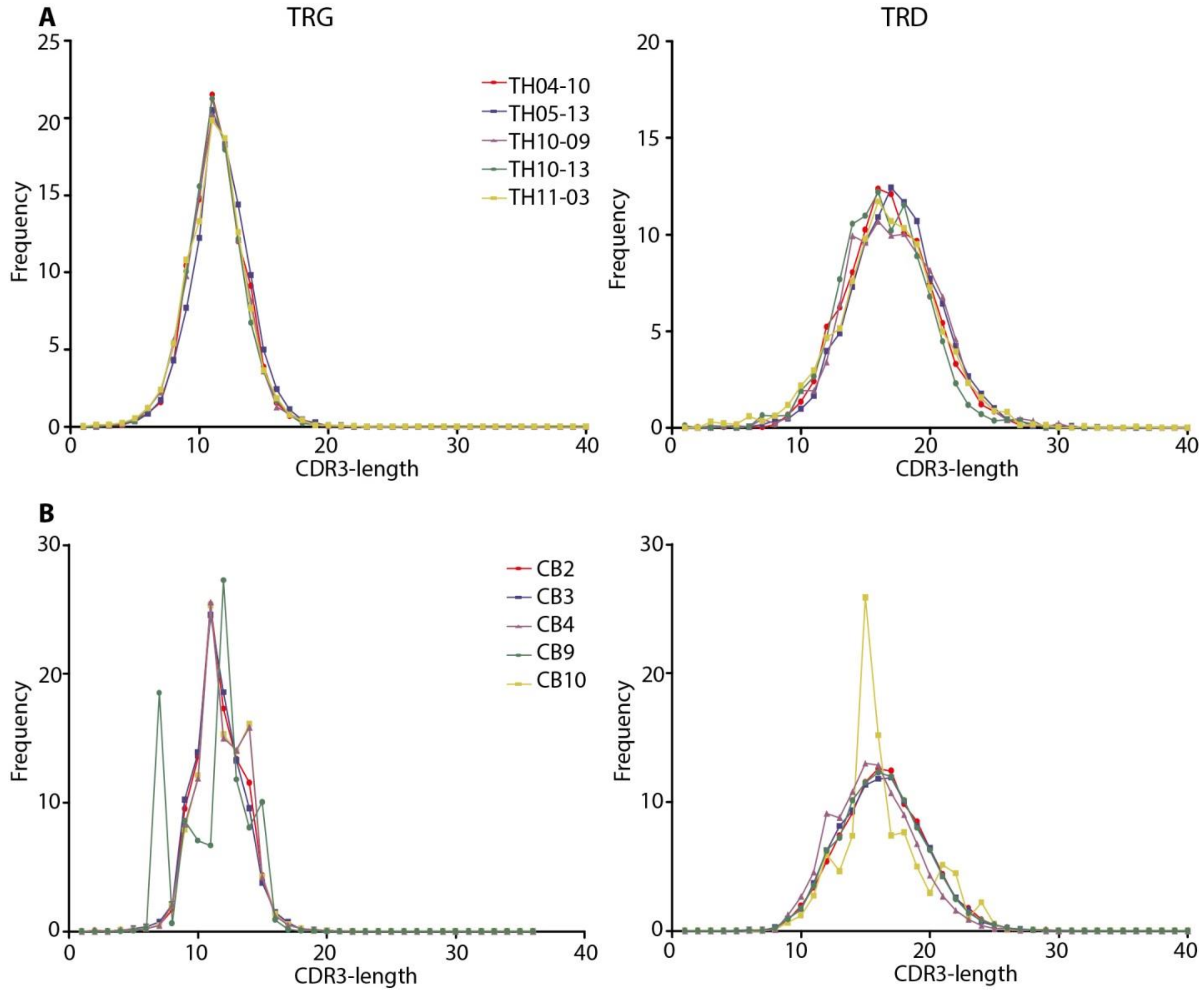

Supplementary Figure 6. TRG and TRD CDR3-length distributions in thymus (Thy) and cord blood (CB) samples. Mean frequencies of four replicates per Thy and CB sample are depicted for TRG and TRD CDR3-regions **(A)**. CB samples showed more prominent peaks in TRG CDR3-lengths, while TRD CDR3-regions showed relatively normal distributions **(B)**. Number of replicates per sample, N=4.

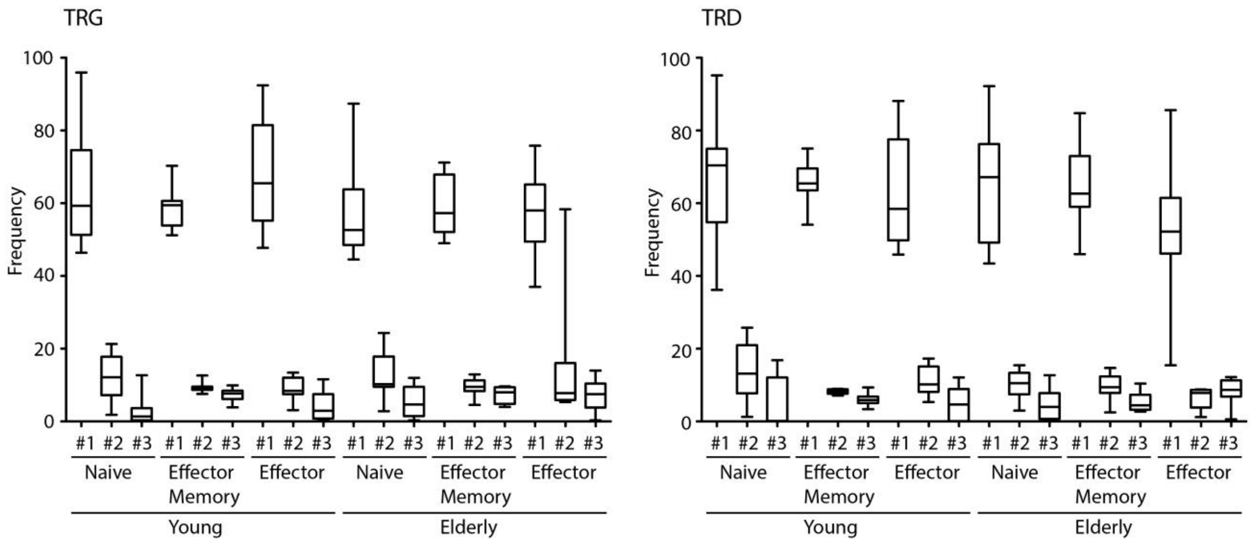

Supplementary Figure 7. Frequencies of sequences coincidences in 1, 2 or 3 PCR replicates per subset and age group. Box-whiskers plots (range 10-90%) depicting numbers of sequences present in either one (#1), two (#2) or three (#3) PCR replicates within all unique sequences based on V-J combination and CDR3 region on nucleotide level. Error bars indicate the SD.
